# The Shift in Synonymous Codon Usage Reveals Similar Genomic Variation during Domestication of Asian and African Rice

**DOI:** 10.3390/ijms232112860

**Published:** 2022-10-25

**Authors:** Guilian Xiao, Junzhi Zhou, Zhiheng Huo, Tong Wu, Yingchun Li, Yajing Li, Yanxia Wang, Mengcheng Wang

**Affiliations:** 1 The Key Laboratory of Plant Development and Environment Adaptation Biology, Ministry of Education, School of Life Science, Shandong University, Qingdao 266237, China; 2 Shijiazhuang Academy of Agriculture and Forestry Sciences, Shijiazhuang 050041, China

**Keywords:** rice, domestication, genomic variation, synonymous codon usage bias, DNA methylation

## Abstract

The domestication of wild rice occurred together with genomic variation, including the synonymous nucleotide substitutions that result in synonymous codon usage bias (SCUB). SCUB mirrors the evolutionary specialization of plants, but its characteristics during domestication were not yet addressed. Here, we found cytosine- and guanidine-ending (NNC and NNG) synonymous codons (SCs) were more pronounced than adenosine- and thymine-ending SCs (NNA and NNT) in both wild and cultivated species of Asian and African rice. The ratios of NNC/G to NNA/T codons gradually decreased following the rise in the number of introns, and the preference for NNA/T codons became more obvious in genes with more introns in cultivated rice when compared with those in wild rice. SCUB frequencies were heterogeneous across the exons, with a higher preference for NNA/T in internal exons than in terminal exons. The preference for NNA/T in internal but not terminal exons was more predominant in cultivated rice than in wild rice, with the difference between wild and cultivated rice becoming more remarkable with the rise in exon numbers. The difference in the ratios of codon combinations representing DNA methylation-mediated conversion from cytosine to thymine between wild and cultivated rice coincided with their difference in SCUB frequencies, suggesting that SCUB reveals the possible association between genetic and epigenetic variation during the domestication of rice. Similar patterns of SCUB shift in Asian and African rice indicate that genomic variation occurs in the same non-random manner. SCUB representing non-neutral synonymous mutations can provide insight into the mechanism of genomic variation in domestication and can be used for the genetic dissection of agricultural traits in rice and other crops.

## 1. Introduction

Cultivated rice is an ancient and widely consumed staple food crop. Two representative cultivated rice species, Asian *Oryza sativa* and African *Oryza glaberrima,* were domesticated from the sympatric Asian wild *O. rufipogon* and African wild *Oryza barthii*, respectively [1,2]. During the domestication process, genome-wide genetic variations occurred, including single nucleotide polymorphisms (SNPs), small insertions and deletions (indels), large size structural variants and so on [3,4,5]. These genome-scale variations provide the genetic basis for the differences in a wide range of morphological and physiological traits between wild and cultivated rice [2,6].

SNPs are the most common and plentiful genetic variations in genomes. SNPs in protein-coding sequences are classified into synonymous and non-synonymous, with much attention paid to the latter; this is because these change peptide sequences and may affect phenotypes. The synonymous SNPs result from the shift in synonymous codons (SCs) that encode the amino acids, except for methionine and tryptophan. The frequencies of SCs encoding a given amino acid are heterogeneous in the genome of a species, resulting in synonymous codon usage bias (SCUB). Nucleotide substitution between SCs does not change the corresponding amino acid residue and is, therefore, often believed to be functionally neutral [7,8]. However, SCUB affects recombination rate, splicing regulation, transcription efficiency, RNA secondary structure, mRNA stability, translational efficiency and accuracy in the regulation of gene expression and protein folding [9,10,11,12,13]. Especially, synonymous mutations in representative yeast genes proved to be strongly non-neutral [14]. Based on these, SCUB may influence mutation rates and the extent of genetic drift and natural selection [15,16,17,18], and is, therefore, an important genetic force for plant evolution. Although genome-wide nucleotide substitutions and other genetic variations occurred during the domestication of rice [3,4,5], whether SCUB shifting played a role in cultivated rice was not studied.

As forms of insertion and deletion (indel), intron gain and loss induce genetic variation and are key evolutionary forces [19,20,21]. The process of indels, such as intron gain and loss, comprises DNA break and repair, which could lead to genomic shock [22,23] and, therefore, induce local single-nucleotide polymorphisms [24,25,26]. As a part of nucleotide substitutions, SCUB in exons is related to adjacent introns in the nuclear genomes [27]. The occurrence of intron gain or loss is associated with both the number of introns and exon position within the gene body [28], so SCUB is reasonably related to these factors. Although the contribution of intron gain and loss in genomic variation during the domestication of rice is still unclear, the number of introns and the exon position were proved to be associated with SCUB shift during plant evolution and somatic hybridization [29,30]. Thus, the relationship between SCUB and the number of introns or exon position following the domestication of rice is worthy of being addressed as it could provide genetic clues for the further understanding of the role of introns in genetic variation.

Apart from genetic variation, the profiles of DNA methylation as a major epigenetic variation altered widely during the domestication of rice [31]. DNA methylation is also a source of genetic variation, because methylated cytosine (5^m^C) can be converted into thymine [32]. Thus, DNA methylation-mediated conversion from cytosine to thymine affects SCUB in plants [29,30]. However, the issues concerning the contribution of DNA methylation to SCUB, as well as the role of SCUB in DNA methylation alteration, were not reported thus far.

In this study, we used Asian and African cultivated rice and their wild species to analyze the characteristics of SCUB during domestication, with the aim of knowing whether domestication can affect SCUB and how DNA methylation contributes to SCUB during domestication. We found that SCUB was obviously affected during the domestication of both Asian and African rice in the same manner, and the dual associations between SCUB and DNA methylation-mediated conversion from cytosine to thymine implies a close link between genetic and epigenetic variations during the domestication process. Our work provides novel data indicating that the SCUB shift possibly provides DNA methylation sites to promote epigenetic variation in the genome during rice domestication; it also demonstrates the bidirectional orchestration between genetic and epigenetic variation.

## 2. Results

### 2.1. C/G-Ending Synonymous Codons Are Preferred in Cultivated Rice

The frequencies of 61 amino acid encoding codons ranged from 0.048 (CGA in *O. glaberrima*) to 0.385 (GAG in *O. glaberrima*) and showed similar patterns in wild and cultivated rice (Figure S1). The frequencies in African wild rice *O. barthii* were slightly distinct from those of the other species, among which A/T-ending codons had higher frequencies but C/G-ending codons lower. Fifty-nine synonymous codons (SCs) encoding eighteen amino acids, except for start codon ATG and AGG encoding Trp, were used for a more detailed analysis. Generally, C/G-ending codons (NNCs and NNGs) were more frequent than A/T-ending codons (NNAs and NNTs) (Figure S2). The patterns of SC frequencies were extremely correlative to RSCU values (Figure S2).

**Figure 1:**
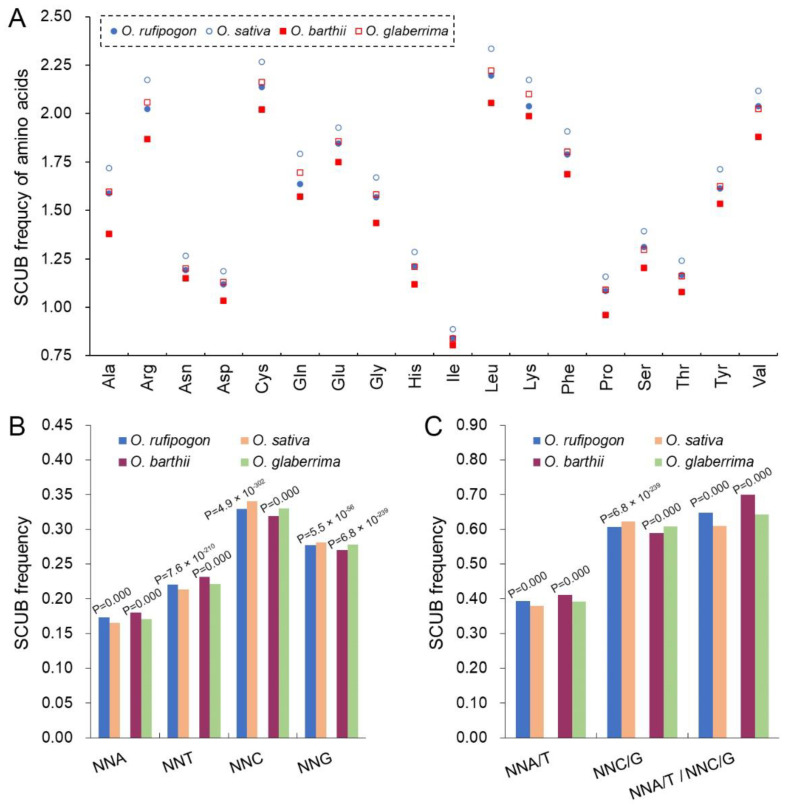
SCUB is heterogeneous between wild and cultivated rice. (**A**): The SCUB frequencies of 18 amino acids, except for Met and Trp, that were defined as the ratios between the number of C/G-ending SCs and A/T-ending SCs. (**B**): The frequencies of NNA, NNT, NNC and NNG codons. NNA, NNT, NNC and NNG: SCs with A, T, C and G as their final base, respectively, N denotes any base. The frequency was calculated as the ratio of the number of all SCs ending with A, T, C or G to the number of all SCs. (**C**): The frequencies of NNA/T and NNC/G codons. NNA/T and NNC/G: SCs with A and T, as well as C and G, as their final base, respectively, N denotes any base. The statistical comparison between wild and cultivated rice was conducted using the Chi-square (χ^2^) test of cross-table analysis.

To directly compare the SCUB of SCs, the SCUB frequency of a given amino acid encoded by SCs, defined as the ratio of the number of C/G-ending SCs (NNCs/Gs) to that of the A/T-ending SCs (NNAs/Ts), was used for analysis. The SCUB frequencies of the 18 amino acids ranged from 0.805 (Ile in *O. barthii*) to 2.335 (Leu in *O. sativa*) (Figure 1A). The SCUB frequencies of the amino acids except for Ile were all higher than 1 (*p* < 1.26 × 10^−45^, Table S1), showing the bias to C/G-ending SCs in both wild and cultivated rice. Moreover, the SCUB frequencies of SCs showed significant differences between cultivated and wild rice, and all 18 amino acids had higher SCUB frequencies in cultivated rice compared with those of wild rice (Figure 1A; Table S2).

SCUB was further evaluated by the total SCUB frequencies of NNA, NNT, NNC and NNG, which were, respectively, defined as the ratios of the number of all NNAs, NNTs, NNCs and NNGs of 59 SCs to the number of the 59 SCs in all the CDS in a genome. In all four species, NNC and NNG were more pronounced than NNA and NNT; NNC frequency was obviously higher than NNG frequency, while NNA frequency was lower than NNT frequency (*p* = 0.000, Figure 1B). The frequencies of NNC/G were higher than those of NNA/T (*p* = 0.000), and the ratios of NNA/T to NNC/G ranged from 0.609 to 0.699 (Figure 1C). The frequencies of NNA, NNT, NNC or NNG codons differed between wild and cultivated rice (*p* = 0.000, χ^2^ test; Figure 1B,C). The frequencies of NNA, NNT and NNA/T were higher in wild rice than in cultivated rice, but the frequencies of NNC, NNG and NNC/G were the converse (*p* = 0.000~5.5 × 10^−56^, χ^2^ test). Moreover, SCUB frequencies were different in Asian and African rice (Figure 1B,C; Table S3). NNA, NNT and NNA/T frequencies in both wild and cultivated Asian rice were correspondingly lower than those in African rice, while NNC, NNG and NNC/G frequencies were the converse. Consistent with SCUB frequencies, the indices such as CAI, CBI and Nc were higher in cultivated rice than in wild rice (Table S4), showing the SCUB frequency used here can reflect the alteration of SCUB during rice domestication. These results indicate that although SCUB is different in Asian and African rice, the bias to NNC/Gs became stronger during the domestication in both Asian and African rice.

We further compared the SCUB frequencies among 12 chromosomes (Figure S3). In both wild and cultivated rice, NNA, NNT and NNA/T frequencies were lower than NNC, NNG and NNC/G frequencies across the 12 chromosomes, similar to the difference based on the whole genome. The frequencies were similar from the first to tenth chromosomes; in the eleventh and twelfth chromosomes, the NNA and NNT frequencies were higher, but the NNC and NNG frequencies were lower, and the difference between NNA/T and NNC/G frequencies decreased. The differences in the frequencies among the 12 chromosomes were similar among all the species (Figure S4). Compared with wild rice, cultivated rice had lower NNA and NNT frequencies but higher NNC and NNG frequencies across the chromosomes. Compared with African rice, Asian rice had higher NNC/G frequencies but lower NNA/T frequencies across the chromosomes, as was consistent with the results based on the whole genome, further confirming the bias to C/G-ending codons during the domestication of rice.

### 2.2. Cultivated Rice Exhibits Stronger Bias to A/T-Ending Synonymous Codons Following the Rise in Intron Number

**Figure 2:**
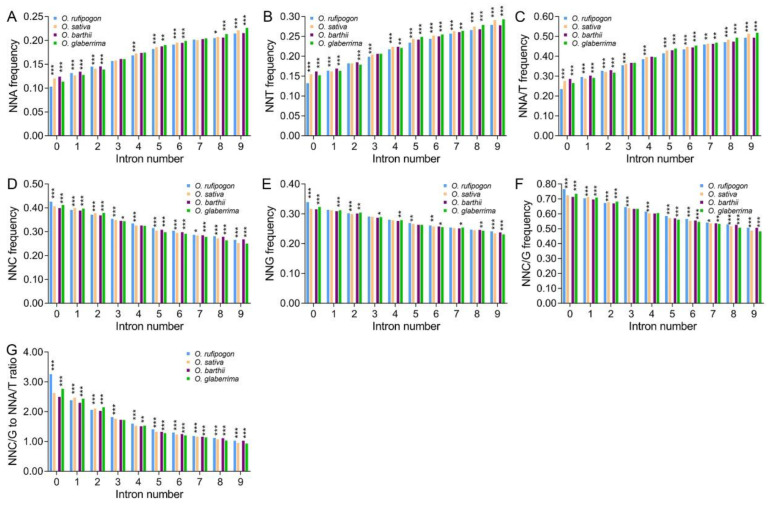
The difference in SCUB frequencies between wild and cultivated rice is associated with intron number. (**A**): The frequencies of A-ending SCs (NNA) in genes with no to nine introns. (**B**): The frequencies of T-ending SCs (NNT) in genes with no to nine introns. (**C**): The frequencies of A- and T-ending SCs (NNA/T) in genes with no to nine introns. (**D**): The frequencies of C-ending SCs (NNC) in genes with no to nine introns. (**E**): The frequencies of G-ending SCs (NNG) in genes with no to nine introns. (**F**): The frequencies of C- and G-ending SCs (NNC/G) in genes with no to nine introns. (**G**): The ratios of C/G-ending SCs to A/T-ending SCs (NNC/G to NNA/T ratios) in genes with no to nine introns. N denotes any base. The difference between wild and cultivated rice was calculated with chi square (χ^2^) test of cross-table analysis (*: *p* < 0.05; **: *p* < 0.01; ***: *p* < 0.001); the results are presented in Table S7.

Intron evolution is a common event in the course of plant evolution and affects SCUB [29,30], so we compared the relation between the SCUB shift frequency and intron number during the domestication of rice. In both wild and cultivated rice, NNC had higher frequencies than NNG in genes harboring no to nine introns, and NNT had higher frequencies than NNA (Figure S5). The frequencies of NNA and NNT gradually increased with the rise in intron number, while the frequencies of NNC and NNG gradually decreased (Figure 2A–D and Figure S5). NNC and NNG frequencies were obviously higher than NNA and NNT frequencies in genes with no or few introns, and the difference became weaker linearly following the rise in intron number. NNT frequencies were even higher than NNG frequencies in genes with more than six introns, and became the highest in genes with nine introns. Consistently, in genes with fewer than nine introns, the frequencies of NNC/G were higher than those of NNA/T (*p* = 1.27 × 10^–4^~0.001, *t*-test); for genes with nine introns, NNA/T and NNC/G were comparable (*p* = 0.377) (Figure 2E,F). Following the rise in intron number, the increase in NNT frequencies was stronger than that of NNA frequencies, and the difference between NNA and NNT frequencies grew; the decrease in NNC frequencies was greater than that of NNG frequencies, and the difference between NNC and NNG frequencies decreased (Figure S5; Table S5).

Compared with wild rice, NNA and NNT frequencies of cultivated rice were lower in genes with no or few introns (except for intronless genes in Asian rice), but higher in genes with more introns (Figure 2A,B; Table S6). For example, the ratios of NNA frequencies between cultivated to wild increased from 0.962 (one intron) to 1.031 (nine introns) in Asian rice, and from 0.917 (no introns) to 1.050 (nine introns) in African rice (Table S6). The difference in NNA frequencies between cultivated and wild rice became more obvious in genes following the rise in intron number (*p* values became smaller, χ^2^ test). Conversely, compared with wild rice, NNC and NNG frequencies in cultivated rice were higher in genes with no or few introns (except for intronless genes in Asian rice), but lower in genes with more introns (Figure 2C,D; Table S6). NNA/T and NNC/G frequencies exhibited similar alteration trends to NNA/NNT and NNC/NNG frequencies, respectively, in genes with no to nine introns between cultivated and wild rice, so that the ratios of NNC/G to NNA/T were larger in genes with no or few introns in cultivated rice than in wild rice (except for intronless genes in Asian rice), but were smaller in genes with more introns (Figure 2E,F; Table S6). These results indicate that following the rise in intron number, the bias to NNA/T became stronger, and the bias appeared to be more drastic in cultivated rice than in wild rice.

### 2.3. Cultivated Rice Has Stronger Bias to A/T-Ending Codons in Internal Exons

**Figure 3:**
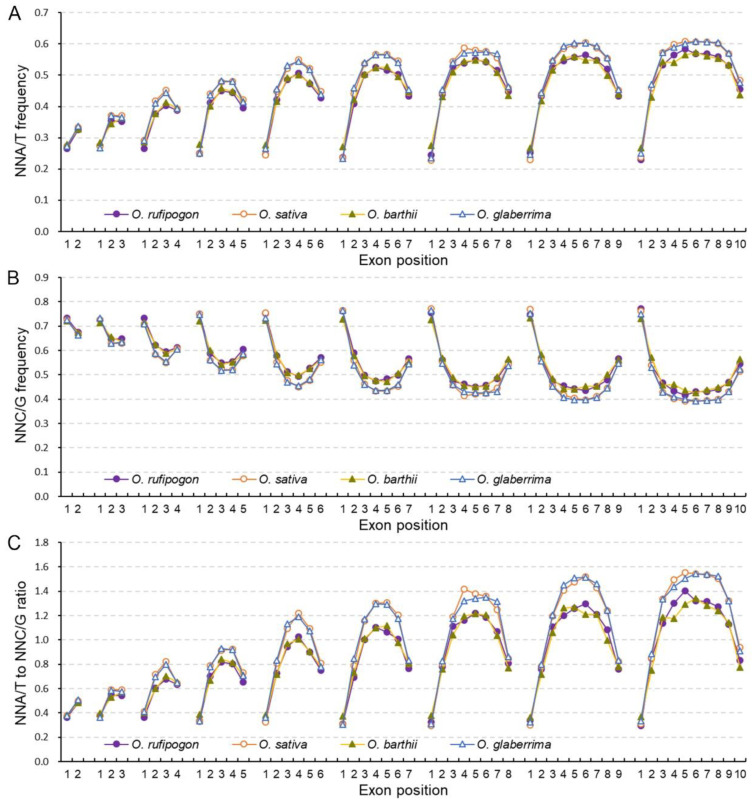
The heterogeneity of SCUB among exons differentiates wild and cultivated rice. (**A**): The frequencies of A- and T-ending SCs (NNA/T) across the exons in genes with two to ten exons. (**B**): The frequencies of C- and G-ending SCs (NNC/G) across the exons in genes with two to ten exons. (**C**): The ratios of A- and T-ending SCs to C- and G-ending SCs (NNA/T to NNC/G ratios) across the exons in genes with two to ten introns. N denotes any base. The difference between wild and cultivated rice was calculated with the chi square (χ^2^) test of cross-table analysis; the results are presented in Table S9.

Given the SCUB patterns based on intron number were different in wild and cultivated rice, the association between SCUB frequency and exon position along the genes was further analyzed. In both wild and cultivated rice, for genes with two to ten exons, the first exons had lower NNA, NNT and NNA/T frequencies but higher NNC, NNG and NNC/G frequencies than the other exons (Figure 3A,B, Figures S6 and S7), resulting in the lowest NNA/T to NNC/G ratios in the first exons (Figure 3C). The frequencies of NNA, NNT, NNC, NNG, NNA/T and NNC/G, as well as the ratios between NNA/T and NNC/G frequencies, were almost comparable in the first exons (CV = 0.010~0.104) (Table S7), showing the SCUB of the first exon remained constant in the genomes of wild and cultivated rice. In the last exons, the frequencies of NNA, NNT and NNA/T were also lower than those of NNC, NNG and NNC/G, but the difference was not as large as that in the first exons. Furthermore, unlike the first exons, the SCUB frequencies did not remain constant across the last exons in genes with two to ten exons (CV = 0.055~0.128). NNA, NNT and NNA/T frequencies gradually increased but NNC, NNG and NNC/G frequencies gradually decreased with the rise in the exon number up to six (CV = 0.046~0.121), and they were comparable in genes with six to ten exons (CV = 0.007~0.042). Thus, the ratios between NNA/T and NNC/G frequencies gradually increased in genes with two to six exons and then remained constant in genes with six to ten exons (Figure 3C). Moreover, in genes with three to ten exons, the frequencies and ratios of the second exons were correspondingly similar to those of the last exons.

In genes harboring four to ten exons, internal exons had higher NNA, NNT and NNA/T frequencies but lower NNC, NNG and NNC/G frequencies compared with terminal (the first, second and last) exons (Figure 3, Figures S6 and S7). In internal exons, NNA, NNT and NNA/T frequencies increased but NNC, NNG and NNC/G frequencies decreased close to the middle exons. Thus, NNC, NNG and NNC/G frequencies formed in the shape of concave curves (“∪”) across the exons, but NNA, NNT and NNA/T frequencies, as well as the ratio between NNA/T and NNC/G frequencies, formed convex curves (“∩”). The curves appeared to be symmetric from the second to the last exons. Moreover, the curve peaks of the NNA, NNT and NNA/T frequencies increased gradually following the rise in exon number, and the increase became quite weak in genes with eight to ten exons; NNC, NNG and NNC/G frequencies showed contrasting patterns (Figures S6 and S7). Across the third to the last-but-one exons, NNA, NNT and NNA/T frequencies were lower than NNC, NNG and NNC/G frequencies in genes with fewer exons, while they became higher in genes with more exons. For internal exons, the shift in NNC and NNT frequencies was more obvious with the rise in exon numbers than in NNG or NNA frequencies. Thus, the differences between NNA and NNT frequencies became larger, while those of NNCs and NNGs became smaller. The profiles of SCUB frequencies based on exon position were similar in wild and cultivated rice, indicating that the stronger bias to A/T-ending codons in the middle exons was maintained during the domestication of rice.

An obvious difference was present in wild and cultivated rice (Figure 3 and Figure S8; Table S8). In the first exons, wild rice had lower NNC, NNG and NNC/G frequencies but higher NNA, NNT and NNA/T frequencies and NNA/T to NNC/G ratios than cultivated rice, while the trend was the converse in the other exons. In both Asian and African rice, the difference in NNA, NNT or NNA/T, as well as in NNC, NNG or NNC/G frequencies, between wild and cultivated rice, was almost constant in the first, second and last exons of the genes with two to ten exons; the difference, however, became larger in the internal exons following the rise in exon number, being much closer to that in the middle exons. On the other hand, the SCUB frequencies across the exons in genes with two to ten exons were almost the same in Asian and African wild rice, and the curves of SCUB frequencies appeared to almost coincide with each other, as was also found between Asian and African cultivated rice. These results indicate that the heterogeneity of SCUB frequencies across exons, as well as the stronger preference for A/T-ending codons in internal exons after domestication, is the same in both Asian and African rice.

### 2.4. SCUB Shift in Cultivated Rice Is Associated with DNA Methylation-Mediated Conversion of Cytosine to Thymine

DNA methylation serves as a source of nucleotide substitution because methylated cytosine (5^m^C) can be converted into thymine [33]. To investigate whether SUCB shift during rice domestication is associated with DNA methylation-mediated nucleotide substitution, we evaluated the frequencies of NNA and NNG with different nucleotides in the second position (conversion of C to T in the antisense strand causes conversion G to A), as well as the frequencies of NNT and NNC with different nucleotides in the first position of the downstream codon (NT|N and NC|N) (conversion of C to T in the sense strand).

**Figure 4:**
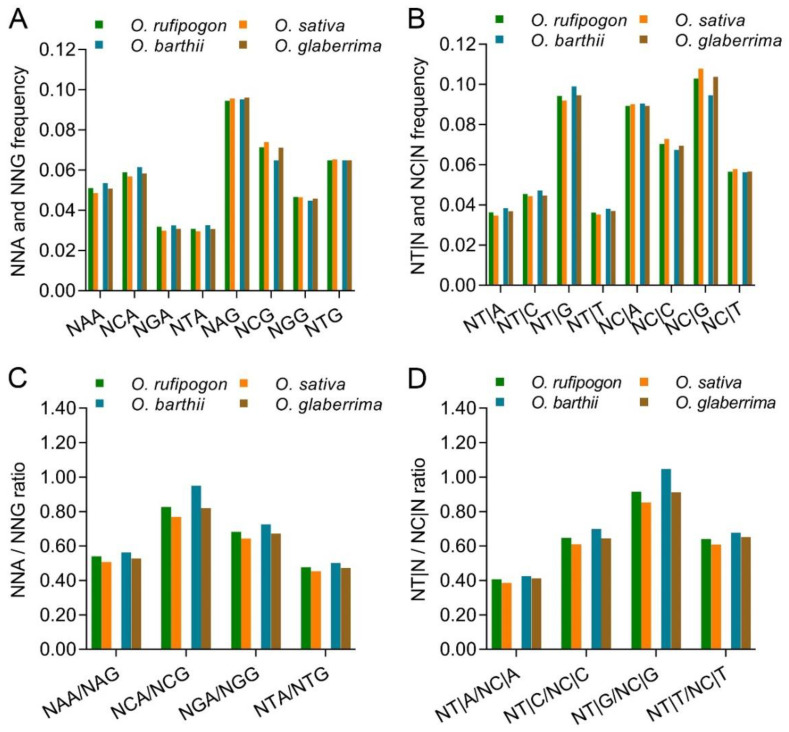
DNA methylation-driven conversion of cytosine to thymine is associated with the difference in SCUB frequencies between wild and cultivated rice. (**A**): The frequencies of NNA and NNG indicating the effect of the second codon nucleotide on the conversion of C to T at the third position in the antisense strand. (**B**): The frequencies of NT|N and NC|N indicating the effect of the first nucleotide of next codons on the conversion of C to T at the third position in the sense strand. (**C**): The ratios of NNA to NNG. (**D**): The ratios of NT|N to NC|N. NNA and NNG: SCs with A and G as the final bases and A, T, C and G at the second position, N denotes any base. NT|N and NC|N: the triple nucleotide combinations with C and T as the final bases and A, T, C and G at the first position of the next codons. The difference in wild and cultivated rice was calculated with chi square (χ^2^) test of cross-table analysis, and the results are presented in Table S10.

Generally, NAA, NCA, NGA and NTA frequencies were slightly lower than NAG, NCG, NGG and NTG frequencies (*p* = 0.055~0.130, *t*-test) (Figure 4A). NCA frequencies were higher than other NNA frequencies; NCG frequencies were lower than NAG, higher than NGG frequencies, but similar to NGG frequencies. NCA/NCG ratios mirroring the methylation-mediated conversion of C to T in the antisense strand were significantly higher than NAA/NAG, NGA/NGG and NTA/NTG ratios (Figure 4C). NNT|G frequencies were drastically higher than NT|A, NT|C and NT|T frequencies and NC|G frequencies were also higher than NC|A, NC|C and NC|G frequencies; this resulted in drastically higher NT|G/NC|G ratios mirroring the methylation-mediated conversion of C to T in the sense strand than for the NT|A/NC|A, NT|C/NC|G and NT|T/NC|T ratios (Figure 4B,D). These data show that C in the second position of the codons and G in the first position of the next codons had a stronger effect in increasing the bias of A and T in the third position of the codons, which indicates the association between methylation-mediated nucleotide conversion and SCUB.

In both Asian and African rice, the frequencies of the four types of NNA codons were lower in cultivated rice than in wild rice; in contrast, among the four types of NNG codons, NCG frequencies were obviously higher in cultivated rice compared with wild rice, but the frequencies of the other three types of NNG codons were substantially less different in the wild and cultivated rice (Figure 4A; Table S9). Thus, the ratios of the four NNA/NNG combinations were lower in cultivated rice than in wild rice, among which the ratios of NCA/NCG had the most pronounced difference between the wild and cultivated rice. Consistently, NT|A, NT|T, NT|C and NT|G frequencies were higher in wild rice than in cultivated rice; for NC|N combinations, NC|G frequencies were significantly lower in wild rice than in cultivated rice, and the difference in the frequencies of NC|A, NC|C and NC|T in wild and cultivated rice was not as remarkable as the NC|G frequencies.

**Figure 5:**
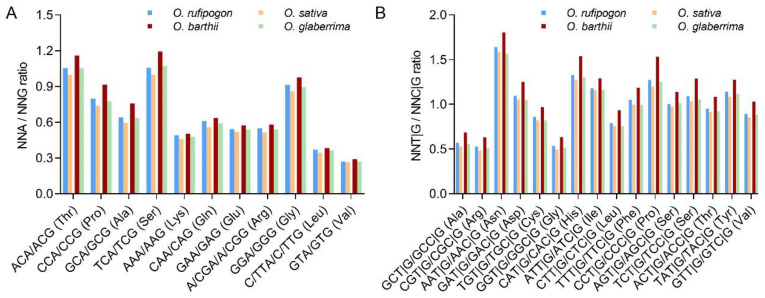
The effect of the adjacent nucleotides on the ratios of A-ending SCs to G-ending SCs, as well as T-ending SCs to C-ending SCs, encoding the given amino acids. (**A**): The effect of the second nucleotide on the codons. (**B**): The effect of the first nucleotide on the next codons. The statistical comparison was conducted using chi square (χ^2^) test, and the results are presented in Table S11. The difference between the ratios of Ala, Pro, Ser, Thr and the ratios of Arg, Gln, Glu, Gly, Leu, Lys and Val in a species was calculated with two-sample *t*-test (*p* < 0.05).

To further analyze the effect of the second nucleotide on DNA methylation-mediated SCUB, the frequencies of C/G-ending SC pairs of amino acids with the same nucleotides in the first and second positions were calculated (Figure 5A). The ratios of NCA/NCG (encoding alanine, proline, serine and threonine) varied from 0.595 to 1.193, significantly higher than those of N(A/G/T)A/N(A/G/T)G (encoding arginine, glycine, glutamic acid, glutamine, leucine, lysine and valine) (0.264 to 0.636 except for glycine (0.858~0.976)) (*p* = 0.009~0.023 and 0.001~0.004 without glycine; *t*-test). Moreover, the ratios of both NCA/NCG and N(A/G/T)A/N(A/G/T)G combinations of these amino acids were lower in cultivated rice than in wild rice (Table S10), and the difference between wild and cultivated rice was more significant in African than in Asian rice (*p* = 0.002 in African rice and 0.501 in Asian rice, *t*-test). On the other hand, the first nucleotide G of the adjacent codons also caused lower ratios of NNT|G/NNC|G in cultivated rice than in wild rice (Figure 5B).

**Figure 6:**
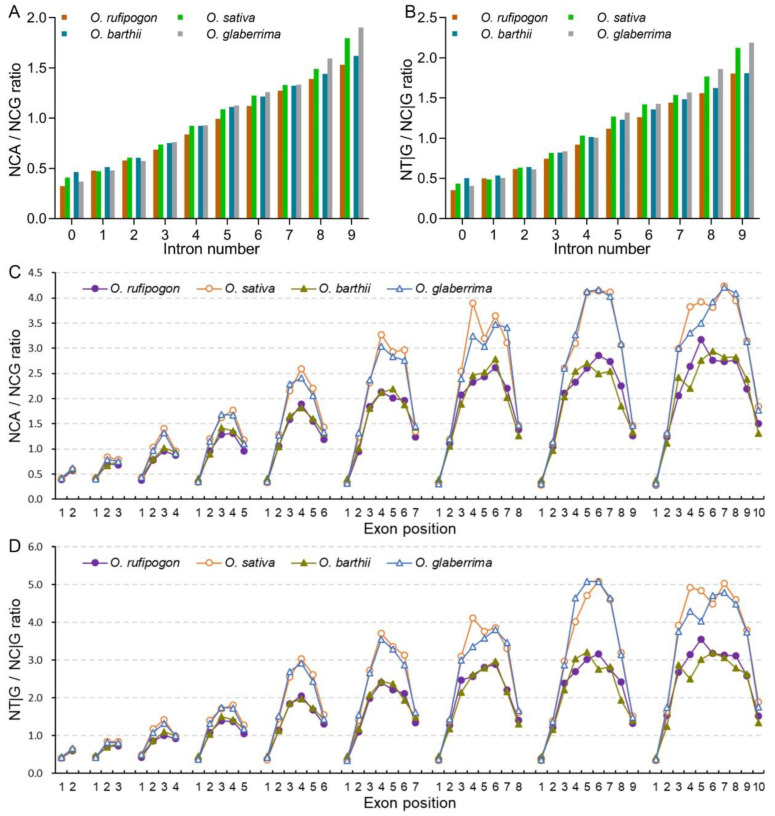
The association between DNA methylation and SCUB heterogeneity based on intron number and exon position reveals the difference between wild and cultivated rice. (**A**): The ratios of NCA to NCG in genes with no to nine introns. (**B**): The ratios of NT|G to NC|G in the genes with no to nine introns. (**C**): The ratios of NCA to NCG across the exons in genes with two to ten exons. (**D**): The ratios of NT|N to NC|N across the exons in genes with two to ten introns. The difference was calculated via chi square (χ^2^) test of cross-table analysis, and the results are presented in Table S12.

The association between DNA methylation and the heterogeneity of SCUB based on introns was further analyzed. The ratios of the four NNA/NNG combinations and the four NT|N/NC|N combinations improved following the increase in intron number (Figure 6A,B and Figure S9). The increase in NCA/NCG and NT|G/NC|G ratios was much sharper than that in the ratios of the other NNA/NNG and NT|N/NC|N combinations (Figure S9), showing DNA methylation-associated SCUB was more preferential in genes containing more introns. Moreover, when compared to wild and cultivated rice, the ratios of both NCA/NCG and NT|G/NC|G were comparable with genes with fewer introns, but were higher in cultivated rice than in wild rice for genes with more introns (Figure 6A,B; Table S11).

The ratios of the four NAA/NNG combinations were almost the same in the first exons of genes with two to ten exons (Figure 6C and Figure S10). In the last exons, the NCA/NCG ratios gradually improved following the increase in the exon number, but the ratios of the other three NNA/NNG combinations were comparable to those in the first exons (Figure S10). The second exons of genes with three to ten exons exhibited similar patterns to the last exons. As for genes with four to ten exons, the ratios of the four NNA/NNG combinations in the internal (the third to the last but one) exons were higher than those in the terminal exons, and gradually increased to be closer to the middle exons, resulting in “∩” curves. The ratios of the four NNA/NNG combinations in the internal exons gradually increased following the rise in exon number, of which the NCA/NCG ratios drastically increased up to more than three in genes with eight to ten exons, but the ratios of NAA/NAG, NGA/NGG and NTA/NTG weakly increased to approximately one (Figure S10). The ratios of NT|A/NC|A, NT|C/NC|C, NT|G/NC|G and NT|T/NC|T among the exons exhibited similar “∩” profiles, and the NT|G/NC|G ratios in the internal exons were more predominant than the other NT|N/NC/N combinations; the increase in the ratios of NT|N/NC|N combinations in the internal exons was more obvious than that of NNA/NNG combinations following the rise in exon number (Figure S11). In both African and Asian rice, the ratios of NAA/NAG, NGA/NGG and NTA/NTG, as well as NT|A/NC|A, NT|C/NC|C and NT|T/NC|T, across the exons in genes with two to ten exons were either comparable to wild and cultivated rice or slightly higher in cultivated rice than in wild rice (Figures S10 and S11). However, the NCA/NCG and NT|G/NC|G ratios in the internal exons were remarkably higher in cultivated rice compared with those in wild rice, and the difference became larger close to the middle exons and more significant following the rise in exon number; the ratios in the first two exons and the last exons were similar in wild and cultivated rice (Figure 6C,D). Moreover, in both wild and cultivated rice, the ratios of the four NNA/NNG and the four NT|N/NC|N combinations across the exons in genes with two to ten exons were similar in African and Asian rice, showing the similar SCUB patterns in wild and cultivated rice.

The association between DNA methylation and SCUB based on exons was further confirmed by C- and G-ending SC pairs of amino acids sharing the same nucleotides in their first and second positions (Figures S12 and S13). The ratios of NCA/NCG combinations were higher than those of N(A/G/T)A/N(A/G/T)G combinations, and the difference became more remarkable following the increase in intron number (Figure S12A–D). Compared with wild rice, cultivated rice had higher ratios of NCA/NCG combinations in genes with more exons (Figure S12E,F). On the other hand, the ratios of NCA/NCG and N(A/G/T)A/N(A/G/T)G combinations among exons exhibited similar “∩” patterns (Figure S13; Table S12). The ratios of the NCA/NCG combinations in internal exons were significantly higher than those of the N(A/G/T)A/N(A/G/T)G combinations; among the N(A/G/T)A/N(A/G/T)G combinations, the ratios of GGA/GGG of glycine in the internal exons were higher than those of the other combinations. The ratios of NCA/NCG combinations in internal exons were obviously higher in cultivated rice than in wild rice, and the difference became more drastic close to the middle exons, as well as following the rise in exon number (Figure S14; Table S13).

### 2.5. SCUB Mirrors the Effect of Domestication

**Figure 7:**
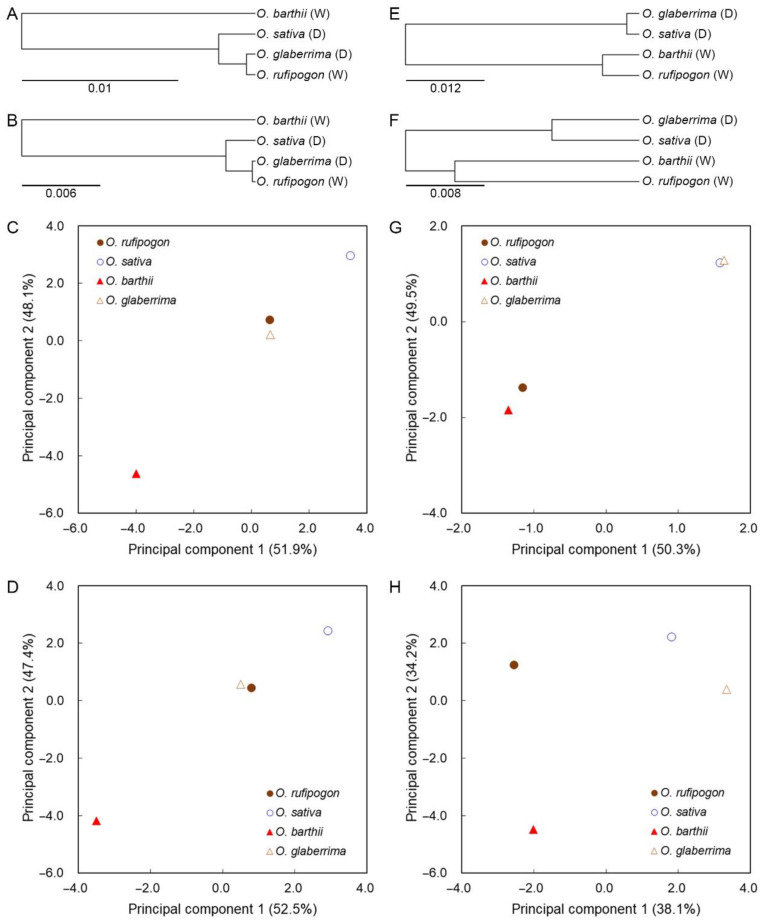
The cluster and principal component analyses of SCUB reflect the domestication of rice. (**A**): A cluster tree based on SCUB frequencies of 59 SCs encoding 18 amino acids. (**B**): A cluster tree based on the ratios of DNA methylation-associated codon combinations. (**C**): A scatter plot of PC1 and PC2 score coefficients from PCA based on SCUB frequencies in 59 SCs encoding 18 amino acids. (**D**): A scatter plot of PC1 and PC2 score coefficients from PCA based on the ratios of DNA methylation-associated codon combinations. (**E**): A cluster tree based on SCUB frequencies across the exons in genes with two to ten exons. (**F**): A cluster tree based on the ratios based on the ratios of DNA methylation-associated codon combinations. (**G**): A scatter plot of PC1 and PC2 score coefficients from PCA based on SCUB frequencies across the exons in genes with two to ten exons. (**H**): A scatter plot of PC1 and PC2 score coefficients from PCA based on the ratios of DNA methylation-associated codon combinations.

Phylogenetic analysis was conducted to outline the association between SCUB and the domestication of rice. The cluster based on both SCUB frequencies and RUSC values of the 59 SCs indicate that African wild rice *O. barthii* and the other three are clustered into two distinct clades, and in the latter clade, African cultivated rice *O. glaberrima* and Asian wild rice *O. rufipogon* are grouped into a sub-clade, differentiated from Asian cultivated rice *O. sativa* (Figure 7A and Figure S15A). This cladistic analysis was confirmed by PCA based on SCUB frequencies and RUSC values (Figure 7C and Figure S15B). The scatter plots of the first and second principal components (PC1 and PC2) distinguish *O. glaberrima* and *O. rufipogon* from *O. barthii* and *O. sativa*. Moreover, in both African and Asian rice, the scatter points of cultivated rice positioned at the top right corner of wild rice, show that SCUB altered in a similar manner during the domestication of Asian and African rice. The cluster using the SCUB frequencies based on exon position and intron number differentiates wild and cultivated rice in a different manner (Figure 7E and Figure S16A). Wild and cultivated rice are clustered into two groups in the PC1–PC2 plot (Figure 7G and Figure S16C). Especially, the PCA using SCUB frequencies based on exon position show that both Asian and African wild rice were close to each other, as were both Asian and African cultivated rice (Figure 7G). These data show that a similar alteration of SCUB in Asian and African rice during their domestication was closely associated with intron. The cluster and PCA using the frequencies of methylation-associated codon combinations obtained similar results to the SCUB frequencies of the 59 SCs (Figure 7B,D), and the analysis using methylation-associated frequencies based on exon position and intron number also differentiated wild and cultivated rice (Figure 7F,H and Figure S16B). Correlation analysis indicates that there are similar correlations with wild and cultivated rice, as well as with Asian and African rice based on the SCUB frequencies of the 59 SCs (Figure S17A–D). As for the SCUB frequencies based on exon position, the correlation of wild and cultivated species was similar between Asian and African rice, and weaker than the correlation between Asian and African wild rice and between Asian and African cultivated rice (Figure S17E–H). Together with the data from the phylogenic tree and the PCA, SCUB appears to reflect the domestication of rice and the association of DNA methylation to SCUB alteration.

## 3. Discussion

As a type of genetic variation, SCUB exhibits diverse profiles in the nuclear genomes of land plants, and can mirror the evolution of plants [29]. Here, both wild and cultivated rice show a preference for C/G-ending SCs (Figure 1). The domestication of wild rice caused a genome-scale genetic variation including nucleotide substitution [3,4,5], and the nucleotide substitution may have changed the SCUB. We found that in both Asian and African rice, the cultivated rice showed more preference for C/G-ending SCs than did wild rice (Figure 1). This demonstrates that domestication indeed affects SCUB and promotes the preference for C/G-ending SCs in cultivated rice. Codon usage bias correlates with the trend of GC content variations [34], so it was proposed that codon usage bias may be driven by GC content changes [35,36]. On other hand, GC-rich regions appear to be prone to homologous recombination, a force of genetic variation, leading to biased gene conversion [37]; this increases the GC content across transcripts [38] and affects codon bias because GC-rich codons tend to be over-represented in ORFs, especially in higher organisms [39]. Thus, homoeologous recombination may partially account for the bias to C/G-ending SCs, and this bias in turn could have promoted homoeologous recombination and genomic variation during the domestication of rice.

Intron gain and loss is a typical genetic event in eukaryotic genomes [40] and causes nuclear substitution in exon sequences; the process is commonly preferential to a lower GC content [41]. Intron-rich genes suffer from stronger selection pressure, so they tend to retain A/T-ending codons [42,43]. Consistent with this, in both wild and cultivated rice, the bias to A/T-ending codons appears to be more pronounced with the rise in intron number (Figure 2). On the other hand, as a type of sequence insertions and deletions (indels), intron evolution could induce nucleotide substitution in adjacent exons, because indels cause nucleotide substitution in several hundred bases of flanking sequences [25,44]. Moreover, a higher bias to A/T-ending codons in internal exons, compared with that in terminal exons, is present in the genes of rice, and the bias in internal exons is more distinguishable in genes with more introns (Figure 3), consistent with the increase in the bias to A/T-ending codons following the increase in the number of introns (Figure 2). These data indicate that the internal exons may be the key point of genetic variation in gene sequences, and are largely responsible for the effect of introns on SCUB. In comparison with wild rice, cultivated rice has higher frequencies of A/T-ending codons in genes with more introns and in the internal exons of the genes (Figure 2 and Figure 3). Especially, the patterns of SCUB frequencies across the exons are almost the same, both between Asian and African wild rice and between Asian and African cultivated rice. Although the association between intron evolution and domestication was not addressed, our findings find new characteristics of genetic variation and suggest that intron evolution may have played an important role in SCUB during the domestication of rice.

Genome-scale DNA methylation was found in both wild and cultivated rice [31]. The methylated cytosine can be converted to thymine [32], so DNA methylation is a source of SNP formation [45]. Consistent with this, the ratios of NCA/NCG and NT|G/NC|G are higher than those of the other NXA/NXG (X = A, G and T) and NT|X/NC|X (X = A, C and T) (Figure 4). Furthermore, the difference in the ratios of both NCA/NCG and NT|G/NC|G in wild and cultivated rice is more pronounced than that of the other NXA/ NXG or NT|X/NC|X (Figure 4, Figure 5 and Figure 6), and coincides with the difference in SCUB frequencies in wild and cultivated rice. This indicates that DNA methylation-driven nucleotide substitution is associated with the SCUB shift in the domestication of rice. In this respect, the ratios of both NCA/NCG and NT|G/NC|G should be higher in cultivated rice than in wild rice. However, these ratios were lower in cultivated than in wild rice (Figure 4). Given that the methylation density and average methylation level of all cytosines in the genome of cultivated rice are higher than those in wild rice [31], it could be speculated that the decrease in DNA methylation-mediated SCUB alteration may result from the conversion of T to C, so as to produce methylation sites, thereby increasing genome-wide DNA methylation levels. On the other hand, the ratios of both NCA/NCG and NT|G/NC|G in the internal exons and intron-rich genes of cultivated rice were higher than those in wild rice (Figure 6). In the genes with body methylation, the internal exons have higher DNA methylation levels than do the terminal exons [46], so introns have a positive effect on DNA methylation-mediated bias to A/T-ending SCs. Therefore, there may be two effects on the association between SCUB and DNA methylation during the domestication of rice: (i) the bias to C- and G-ending SCs contributes to a higher DNA methylation level, and (ii) the higher DNA methylation level results in the bias to A and T-ending SCs by DNA methylation, driving C to T conversion. The synonymous variation seems to be a nonrandom event to orchestrate the domestication and evolution of plants. This is an interesting point to be investigated in the future. Moreover, epigenetic variation such as DNA methylation governs the balance of gene expression [47]. Given the role of SCs in transcription efficiency, mRNA stability, translational efficiency and accuracy [9,10,11,12,13], a shift in SCUB may be detrimental to the phenotype of cultivated rice. Thus, the substitution between SCs can also be used for mining genes and excellent allelic variation governing agricultural traits in rice and other crops.

In summary, our work found that SCUB shifted during the domestication of rice, and that the shift in SCUB exhibits similar characteristics in Asian and African rice, as illustrated by cluster analysis and PCA (Figure 7); this indicates that SCUB and genetic variation is not a random event and provides a new insight into the genomic variation during domestication. Nucleotide substitution polymorphism is an important genetic force in plant evolution and crop improvement. Given the non-neutral effect of synonymous codons within the cells [9,10,11,12,13,14], SCUB may have a detrimental effect on the improvement of agricultural traits in crops; it is, therefore, necessary to focus more attention on the genetic dissection of agricultural traits in the future.

## 4. Materials and Methods

### 4.1. Genome Sequences and Codon Count

Asian wild rice *Oryza rufipogon* and cultivated rice *Oryza sativa*, and African wild rice *Oryza barthii* and cultivated rice *Oryza glaberrima*, were used for analysis. Their genome sequences were downloaded from the EnsemblPlants database (http://plants.ensembl.org/info/data/ftp/index.html (accessed on 16 April 2021)). The coding sequences (CDS) of annotated genes were extracted according to the GFF3 gene-annotation files that were also downloaded from the EnsemblPlants genome database. For genes with more than one transcript type, the first transcript sequence was used for analysis. Any extracted CDS without a length that was a multiple of three, containing N, with start codon not ATG, stop codons not TAA, TAG and TGA were excluded. Codons interrupted by an intron between the first and the second nucleotide were treated as belonging to the downstream exon, while those interrupted between the second and the third nucleotides were deemed to belong to the upstream exon.

### 4.2. Calculation of SCUB Indices

Using CodonW 1.4.2 software (https://sourceforge.net/projects/codonw/ (accessed on 27 February 2013)), all filtered coding sequences (CDS) in the genome of a species were used to calculate the relative synonymous codon usage (RSCU), codon adaptation index (CAI) and other indices of SCUB.

### 4.3. Calculation of SCUB Frequency

We adopted SCUB frequencies to measure the bias of SCs. The frequency of each of the 61 amino acid-encoding codons was calculated using the ratio of the number of this codon to the number of all codons of the filtered CDS in a species. In total, 59 SCs encoding 18 amino acids, except for Met and Trp, were used to calculate SCUB frequency. The SCUB frequency of an amino acid encoded by SCs was defined as the ratio of the number of C- and G-ending SCs to the number of A- and T-ending SCs of this amino acid. Total SCUB frequency was defined as the ratio of the number of all SCs with A, T, C or G at the third position (abbreviated as NNA, NNT, NNC or NNG) to the number of all codons represented in the filtered CDS, except for start codon, stop codons and TGG.

Methylated cytosine (5^m^C) can be converted into thymine [33]; methylation is mainly present in the C of CpG, so the conversion of 5^m^C results in TpG in the sense strand and CpA in the antisense strand. The conversion of NCG to NCA (the second to third position) and NC|G to NT|G (the third-next codon’s first position) can lead to the bias to A- and T-ending codons. Thus, the ratios of the NXA number to the NXG number (X = A, T, C, or G) can indicate the effect of the second nucleotide on the conversion from G and C to A and T at the third position, respectively; in addition, the ratios of the NG|X number to the NC|X number (X = A, T, C, or G) can indicate the effect of the first nucleotide of the next codon on the conversion from G and C to A and T at the third position, respectively. Based on this, the association between DNA methylation and SCUB was evaluated by comparing the difference in the NCA/NCG ratio with the NAA/NAG, NGA/NGG and NTA/NTG ratios, and the difference in the NT|G/NC|G ratio with the NT|A/NC|A, NT|C/NC|C and NT|T/NC|T ratios.

### 4.4. Cluster Analysis and Principal Component Analysis

Employing the average linkage method and the distance measurement of correlation in Minitab 17 statistical software, cluster analysis was performed using the SC frequencies and RSCU values of 59 SCs, the SCUB frequencies based on exon position, the number of introns and the frequencies of codon combinations associated with DNA methylation. The dendrogram was generated on the basis of similarity. The data for cluster analysis were also subjected to principal component analysis in JMP 13 software with default parameters. The factor score coefficients given by the first two principal components were used to generate the scatter plot diagrams.

### 4.5. Statistical Analysis

The difference in the frequencies of NNA, NNT, NNC and NNG of a species and their difference in wild and cultivated rice were calculated using the chi square (χ^2^) test of the cross-table analysis, and the NNA, NNT, NNC and NNG numbers were used for the calculations. The same statistical analysis was performed to compare the difference in the frequencies of NNA/T (A- and T-ending SCs) and NNC/G (C- and G-ending SCs). The χ^2^ test of the cross-table analysis was conducted to evaluate the difference in SCUB frequency related to the third nucleotide position concerning DNA methylation, where the difference between NCA/NCG and NXA/NXG ratios (X = A, G or T) was analyzed using the numbers of NCA, NCG, NXA and NXG; the difference between NC|G/NG|G and NC|X/NG|X ratios (X: A, C, or T, respectively) was analyzed using the numbers of NC|G, NG|G, NC|X and NG|X. The difference between NXC and NXG (X: A, C, G or T, respectively) SCs of an amino acid encoding by G- and C-ending SCs was measured with the χ^2^ test using the numbers of NXC and NXG. The difference in the SCUB frequencies, based on intron number and exon position in wild and cultivated rice, was calculated via the two-sample *t*-test. Fluctuation was assessed by the coefficient of variation (CV), which was calculated as the ratio of standard deviation to mean.

## Data Availability

Not applicable.

## Supplementary Materials

The following supporting information can be downloaded at: https://www.mdpi.com/article/10.3390/ijms232112860/s1.
