# Supplementary material for: The Shift in Synonymous Codon Usage Reveals Similar Genomic Variation during Domestication of Asian and African Rice
Source: Int J Mol Sci. 2022 Oct 25;23(21):12860. doi: 10.3390/ijms232112860 (PMC9656316; doi:10.3390/ijms232112860)
Supplement: Supplementary file 1 [file ijms-23-12860-s001.zip › Supp figures.pdf]

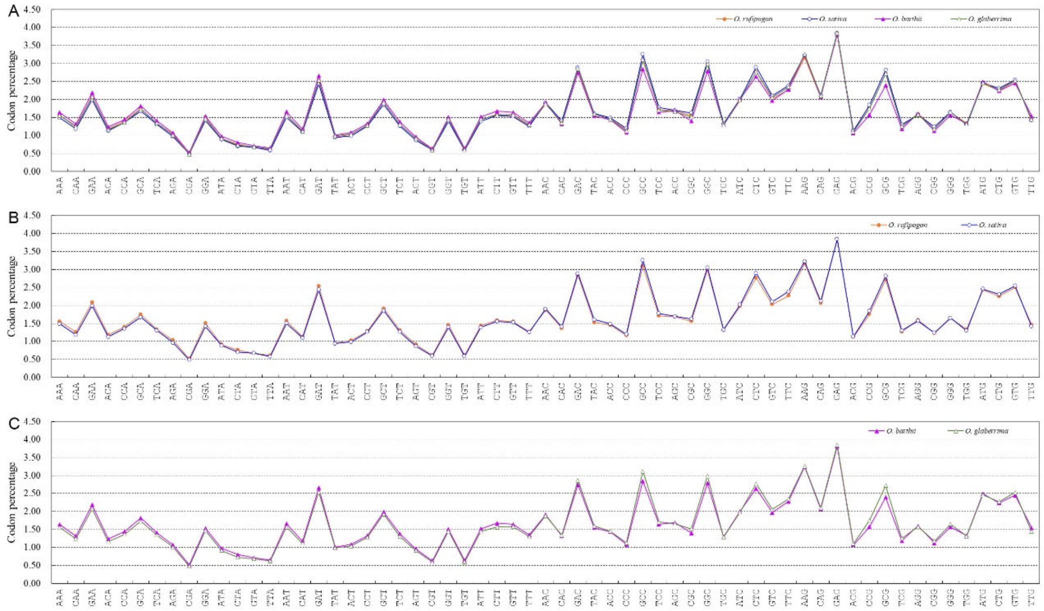

Figure S1. The frequencies of 61 amino acid encoding codons in the genomes of Asian and African rice. A: The frequencies of four species. B: The frequencies of Asian rice. C: The frequencies of African rice. The frequency was defined as the number of each codon to the number of total 61 codons except for three stop codons.

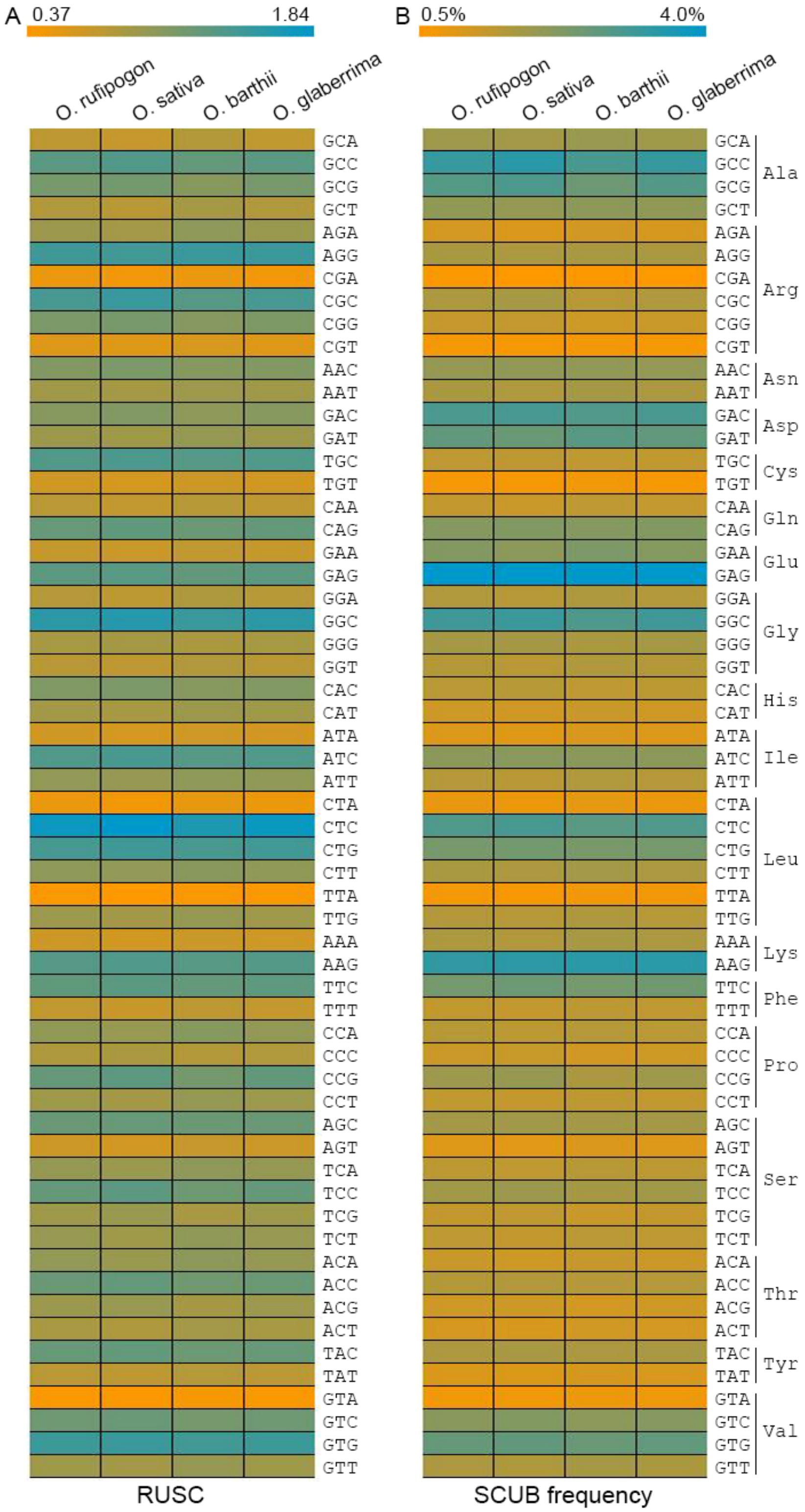

Figure S2. The comparison with RSCU values and SCUB frequencies of 59 SCs encoding 18 amino acids. RSCU values were calculated with CodonW software. SCUB frequency was calculated by the ratio of the number of each SC to the number of total SCs.

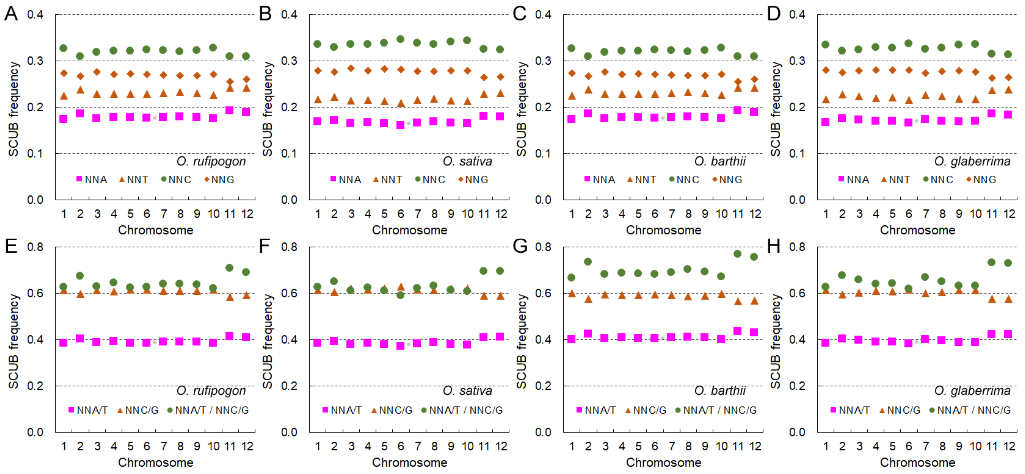

Figure S3. The SCUB frequencies in 12 chromosomes of rice. A-D: The frequencies of NNA, NNT, NNC and NNG codons in four species respectively. NNA, NNT, NNC and NNG: SCs with A, T, C and G as their final base respectively, N denotes any base. The frequency was calculated as the ratio of the amounts number of all SCs ending with A, T, C or G to the amount of all SCs. E-H: The frequencies of NNA/T and NNC/G codons as well as their ratios in four species respectively. NNA/T and NNC/G: SCs with A and T or C and G as their final base respectively, N denotes any base. The frequency was calculated as the ratio of the amounts number of all SCs ending with A and T or C and G to the amount of all SCs.

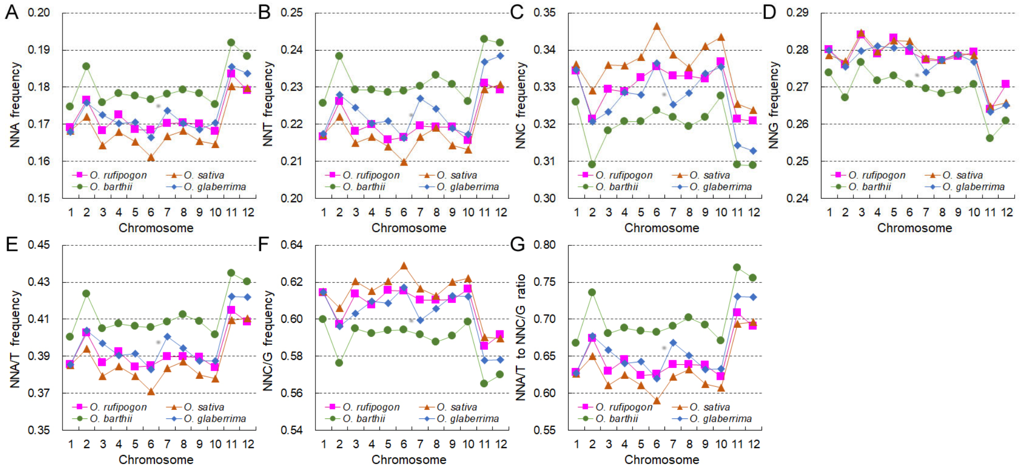

Figure S4. The comparison of the SCUB frequencies in 12 chromosomes among four rice species. A-D: The comparison of the frequencies of NNA, NNT, NNC and NNG codons respectively. NNA, NNT, NNC and NNG: SCs with A, T, C and G as their final base respectively, N denotes any base. The frequency was calculated as the ratio

of the amounts number of all SCs ending with A, T, C or G to the amount of all SCs. E-G: The comparison of the frequencies of NNA/T and NNC/G codons as well as NNA/T to NNC/G ratios respectively. NNA/T and NNC/G: SCs with A and T or C and G as their final base respectively, N denotes any base. The frequency was calculated as the ratio of the amounts number of all SCs ending with A and T or C and G to the amount of all SCs.

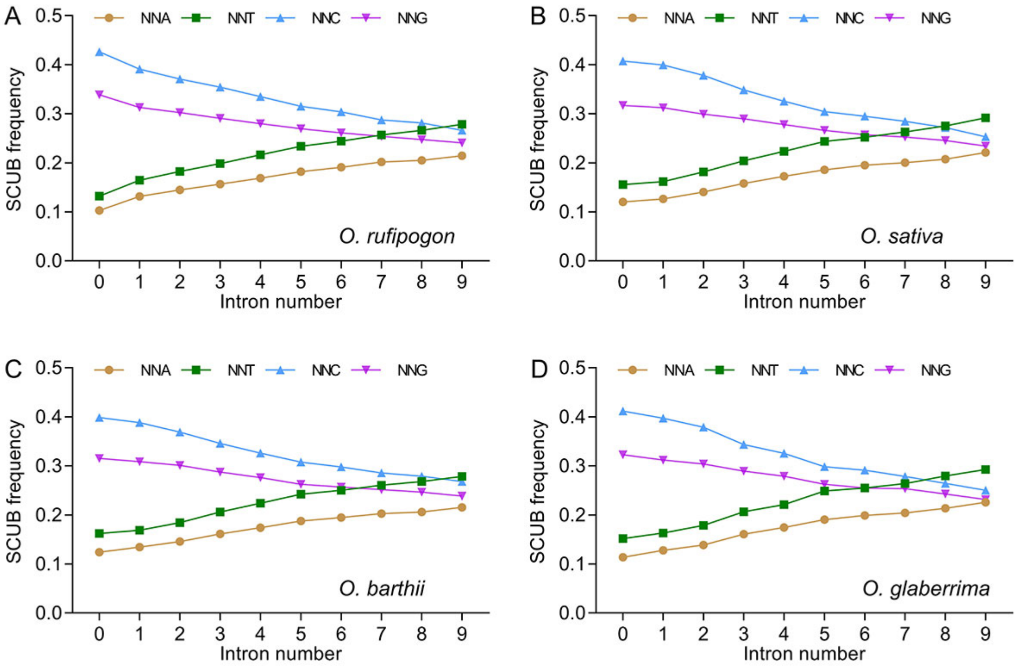

Figure S5. The SCUB frequencies of genes containing none to nine introns. A: Asian wild rice. B: Asian cultivated rice. C: African wild rice. D: African cultivated rice. NNA, NNT, NNC and NNG: SCs with A, T, C and G as their final base respectively, N denotes any base.

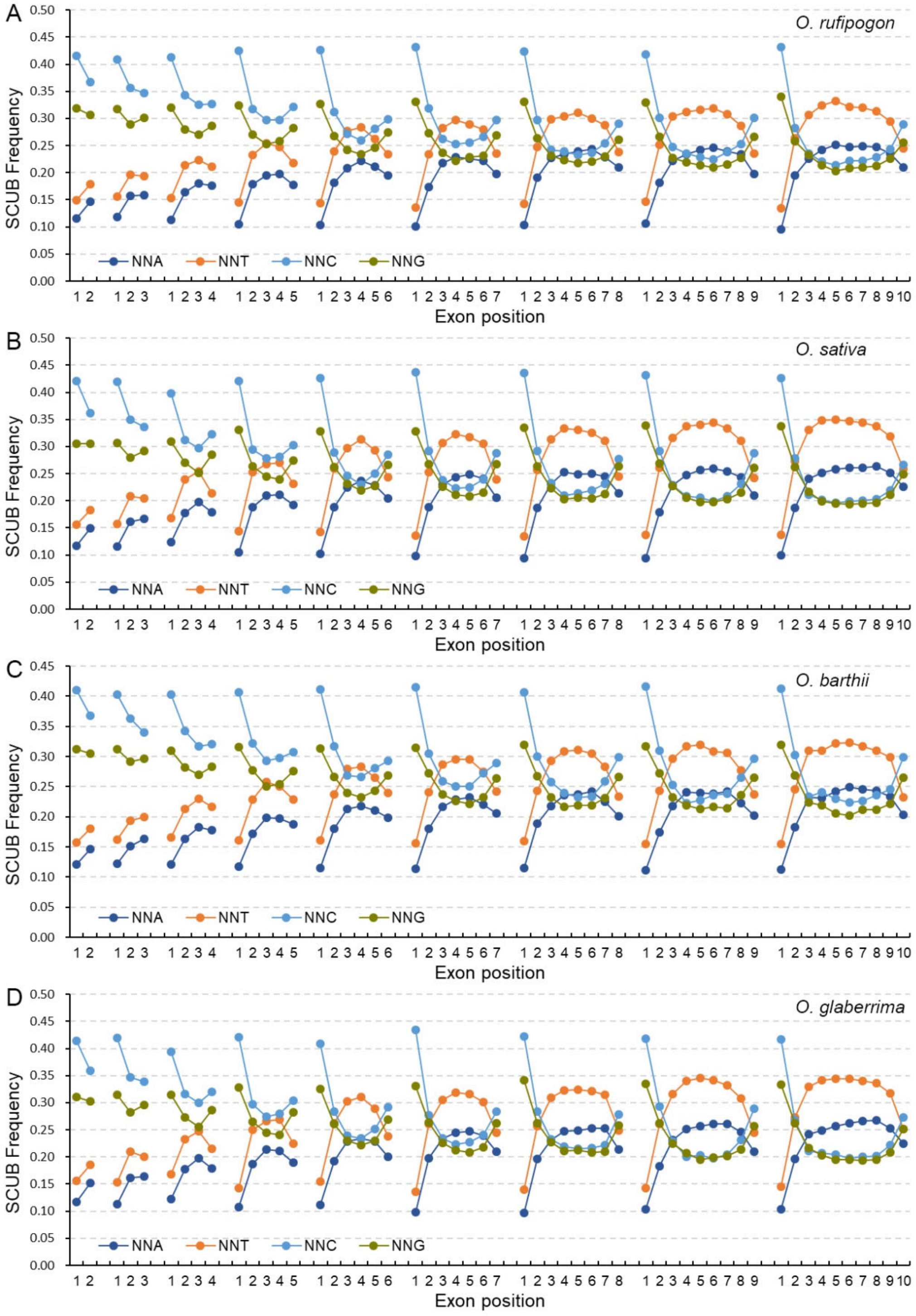

Figure S6. The frequencies of A, T, C and G-ending codons with across exons in genes with two to ten exons. A: Asian wild rice. B: Asian cultivated rice. C: African wild rice. D: African cultivated rice. NNA, NNT, NNC and NNG: SCs with A, T, C and G as the third base respectively, N denotes any base.

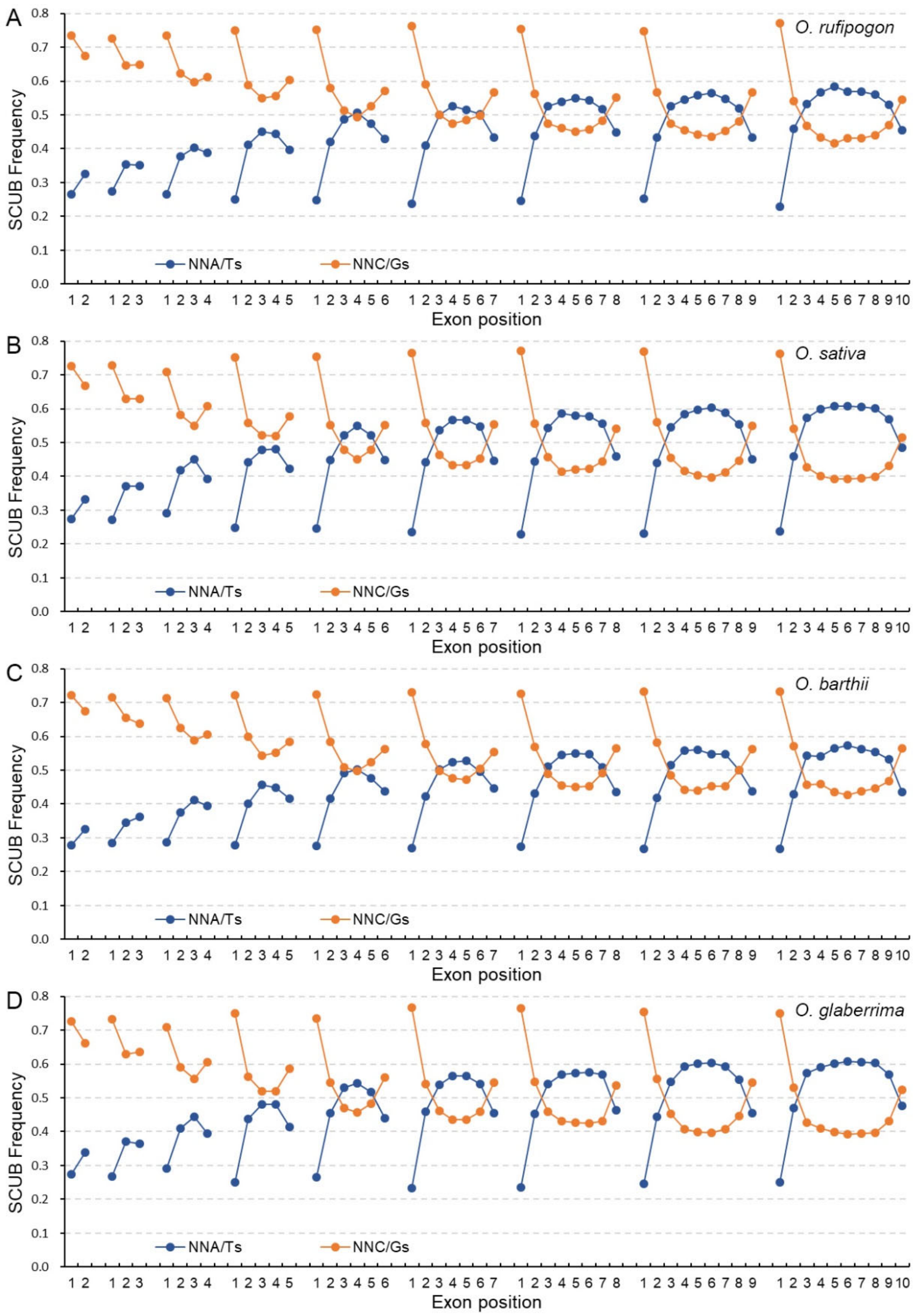

Figure S7. The frequencies of A/T and C/G-ending codons across exons in genes with two to ten exons. A: Asian wild rice. B: Asian cultivated rice. C: African wild rice. D: African cultivated rice. NNA/T, and NNC/G: SCs with A and T or C and G as their final base respectively, N denotes any base.

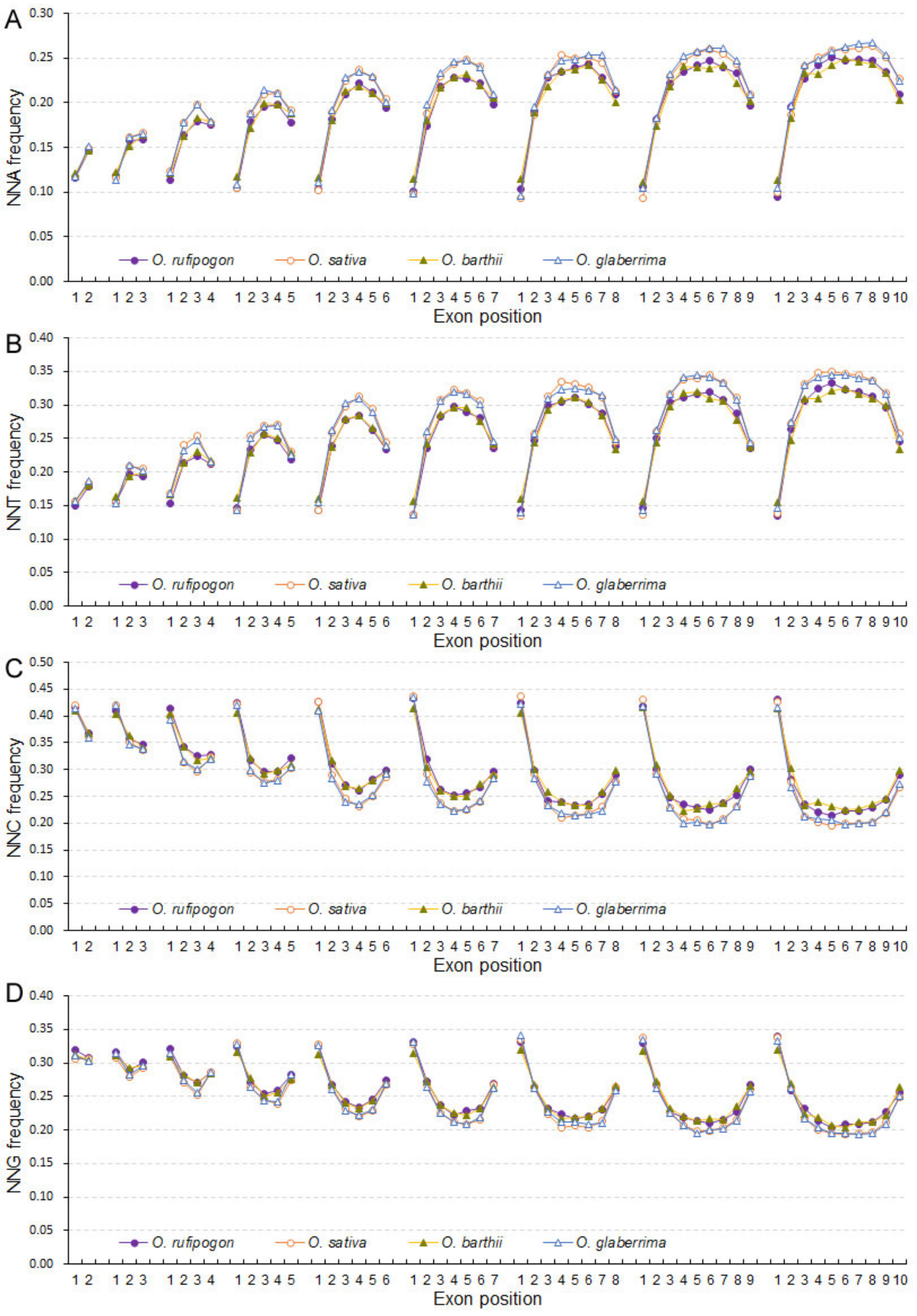

Figure S8. The comparison of SCUB frequencies across exons in genes with two to ten exons among rice species. NNA, NNT, NNC and NNG: SCs with A, T, C and G as the third base respectively, N denotes any base.

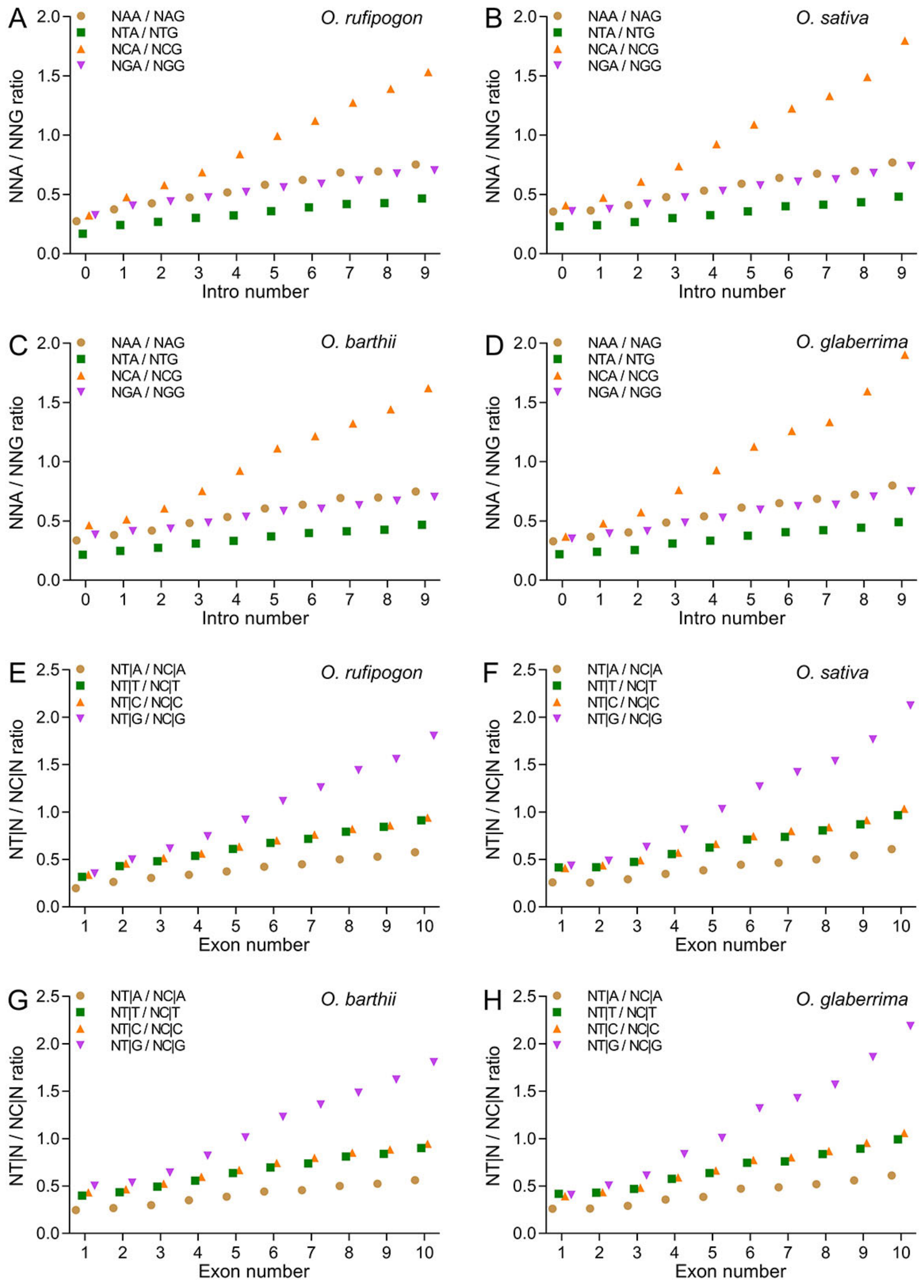

Figure S9. The association between DNA methylation and SCUB in genes with none to nine introns. A-D: The effect of the second nucleotide on the bias of A and G at the third position. E-H: The effect of the first nucleotide of the next codons on the bias of T and C at the third position.

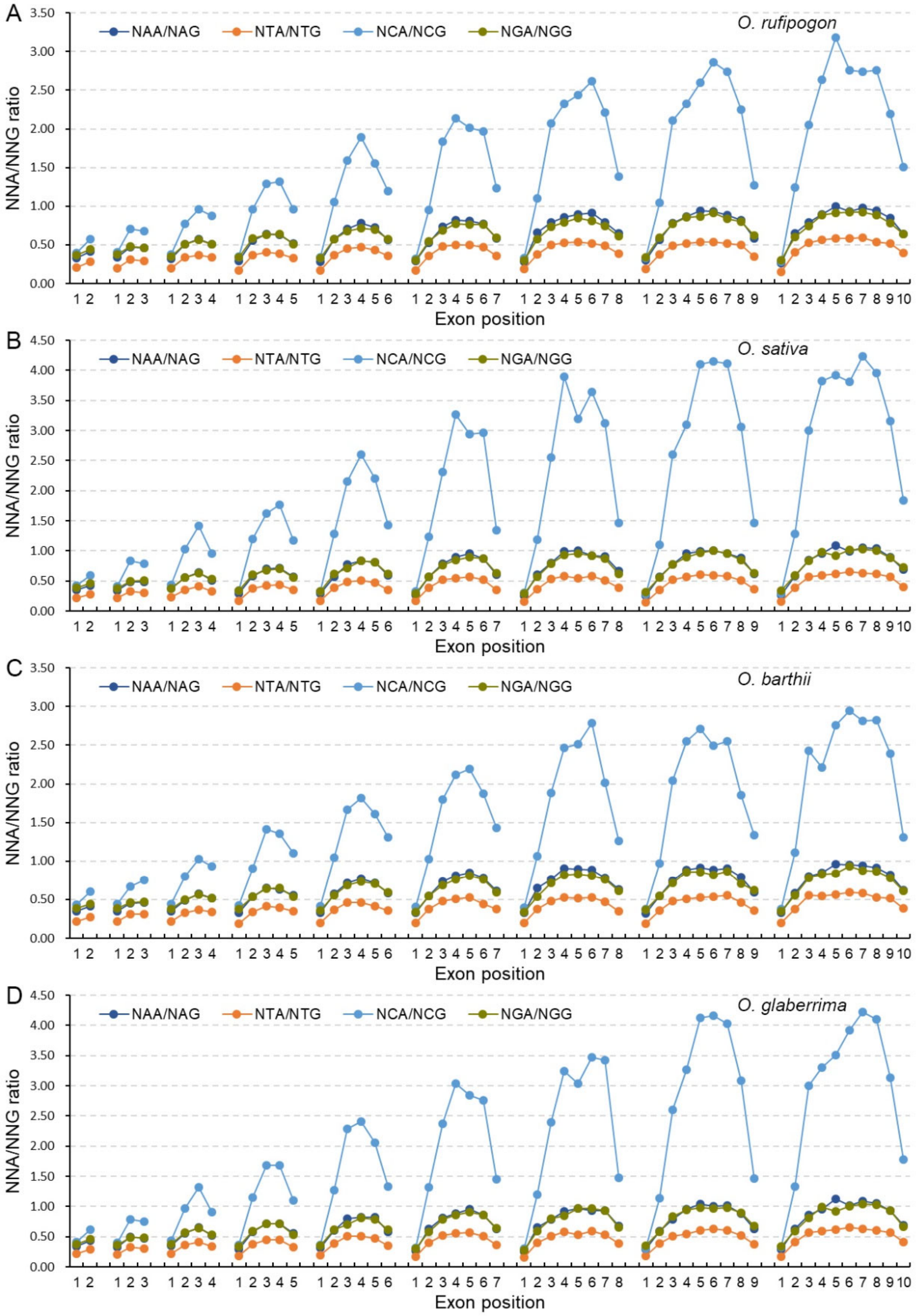

Figure S10. The association between DNA methylation and SCUB across exons in genes with two to ten exons indicated by the effect of A, T, C and G at the second position on the bias of A and G at the third position.

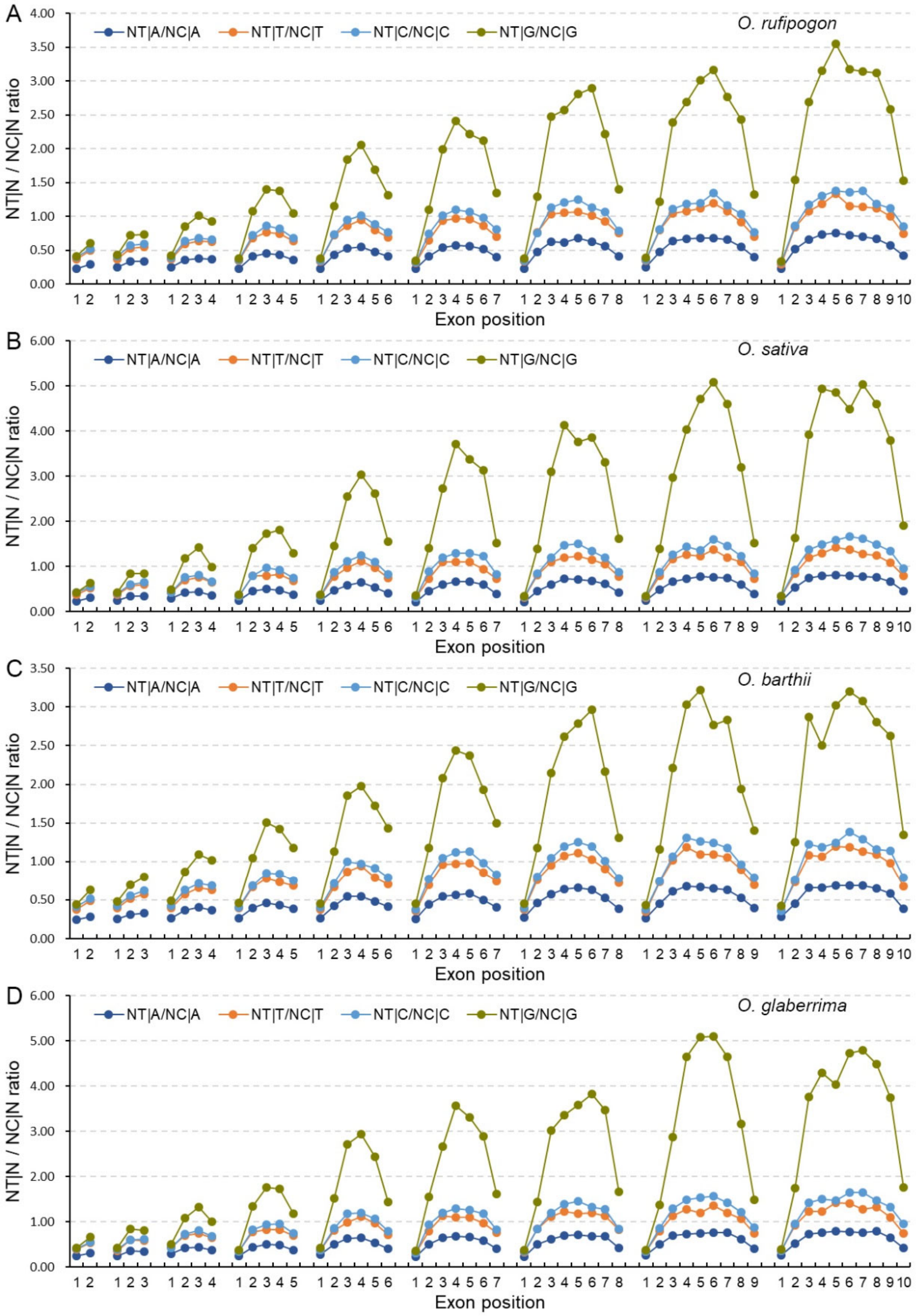

Figure S11. The association between DNA methylation and SCUB across exons in genes with two to ten exons indicated by the effect of A, T, C and G at the first position of the next codons on the bias of T and C at the third position.

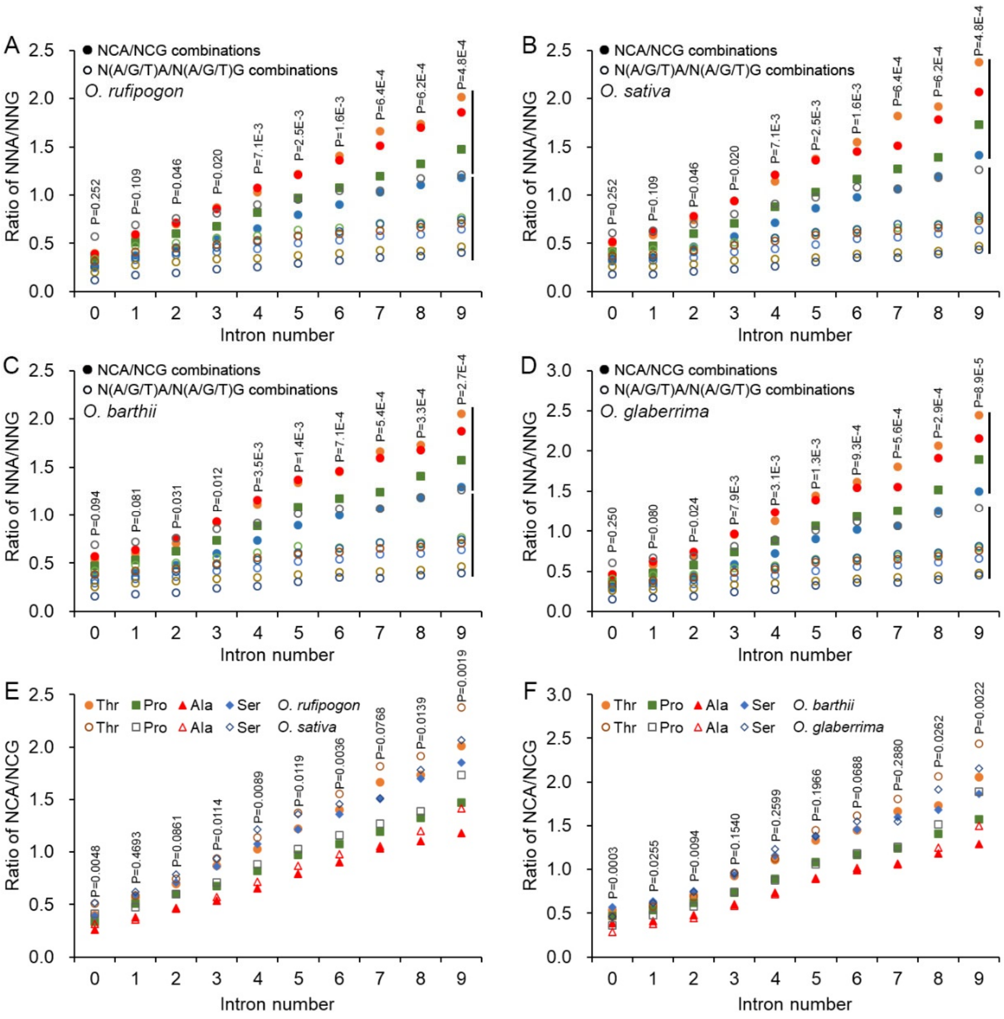

Figure S12. The ratios of A-ending SCs to G-ending SCs encoding the given amino acids in genes with none to nine introns. A-D: The ratios in four rice species. E, F: The comparison of NCA/NCG combination between wild and cultivated rice. NCA/NCG combination: the ratios of NCA SCs to NCG SCs with C at the second position encoding Ala, Pro, Ser and Thr. N(A/G/T)A/N(A/G/T)G combination: the ratios of NNA SCs to NNG SCs with A, G and T but not C at the second position encoding Arg, Gln, Glu, Gly, Leu and Lys.

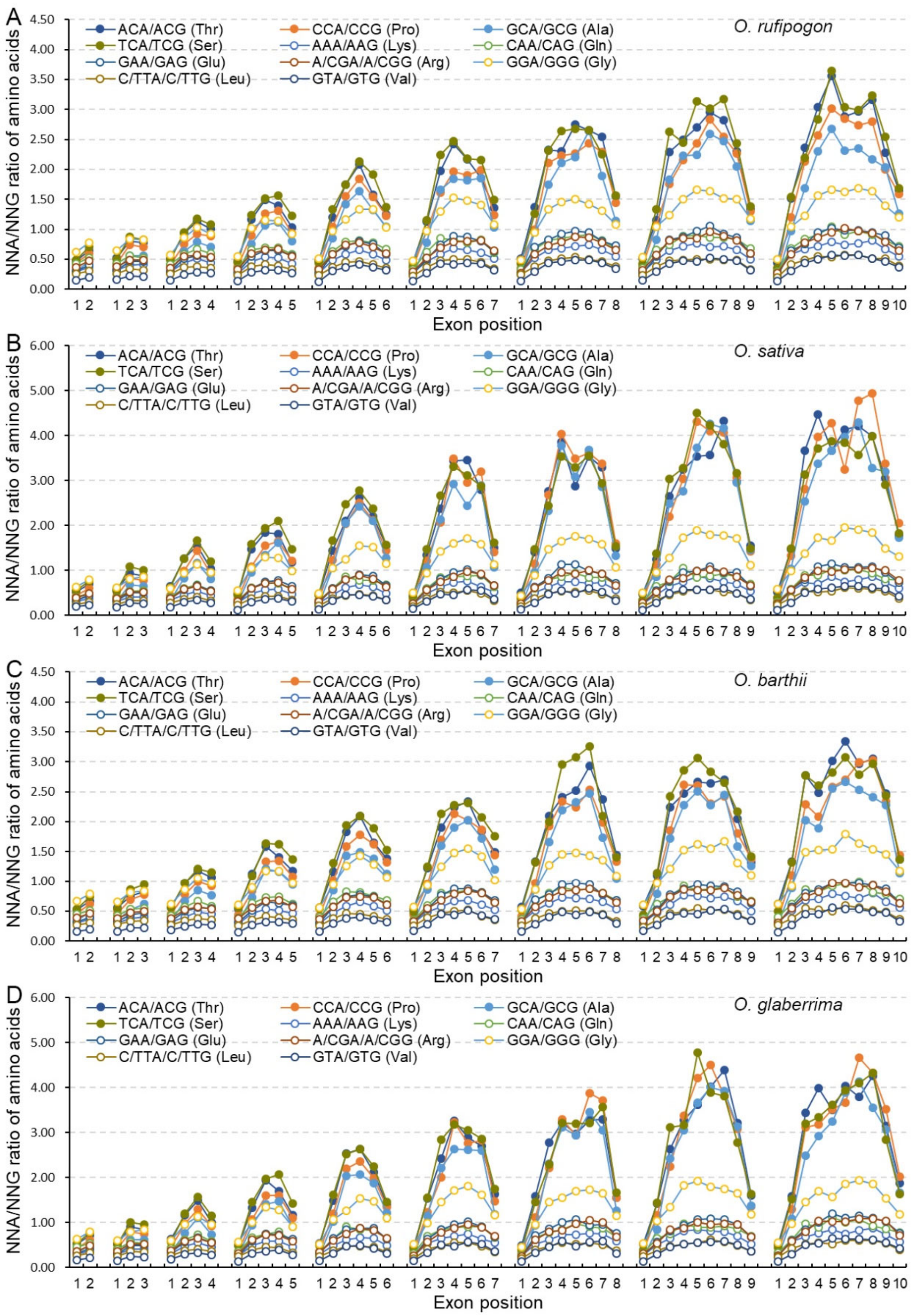

Figure S13. The ratios of A-ending SCs to G-ending SCs encoding the given amino acids across exons in genes with two to ten exons.

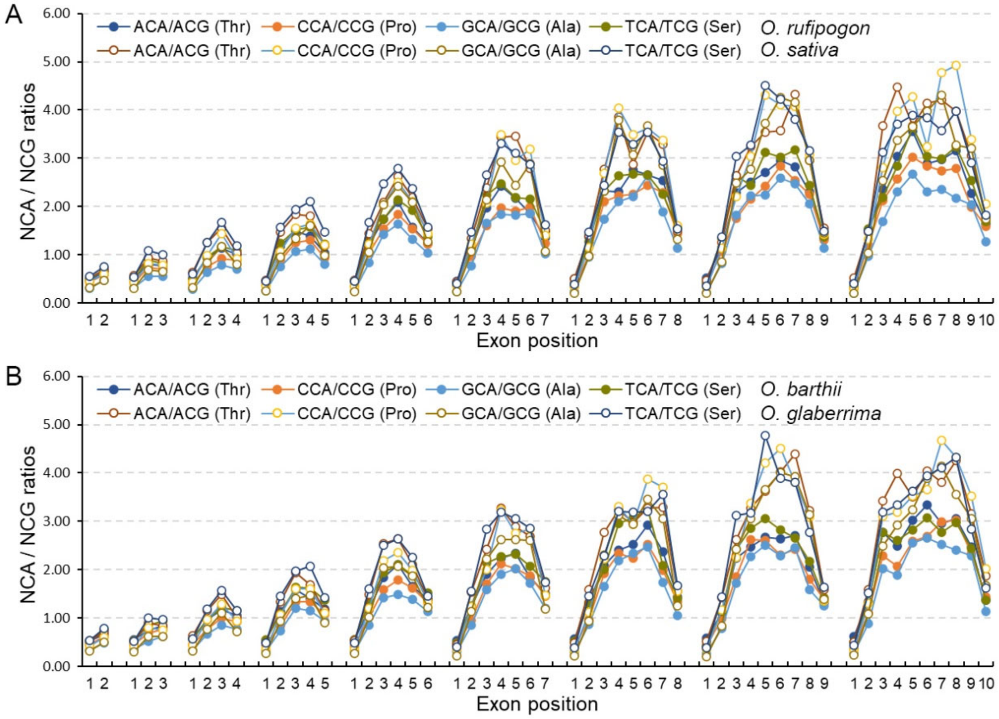

Figure S14. The comparison of the ratios of A-ending SCs to G-ending SCs encoding the given amino acids across exons in genes with two to ten exons between wild and cultivated rice.

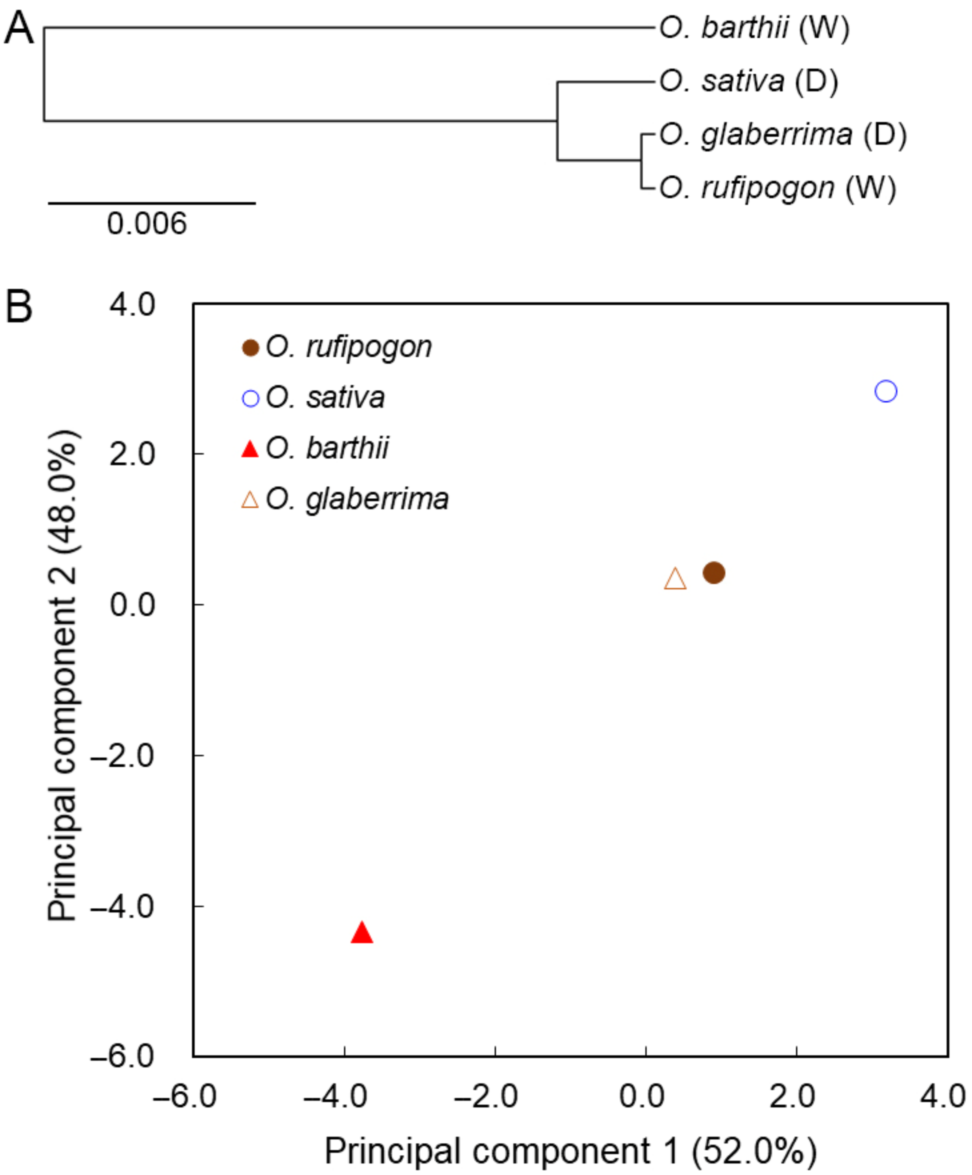

Figure S15. The cluster and principal component analyses using RUSC values. A: A cluster tree based on RUSC values of 59 SCs encoding 18 amino acids. B: The two dimensional scatter plot based on PC1 and PC2 score coefficients.

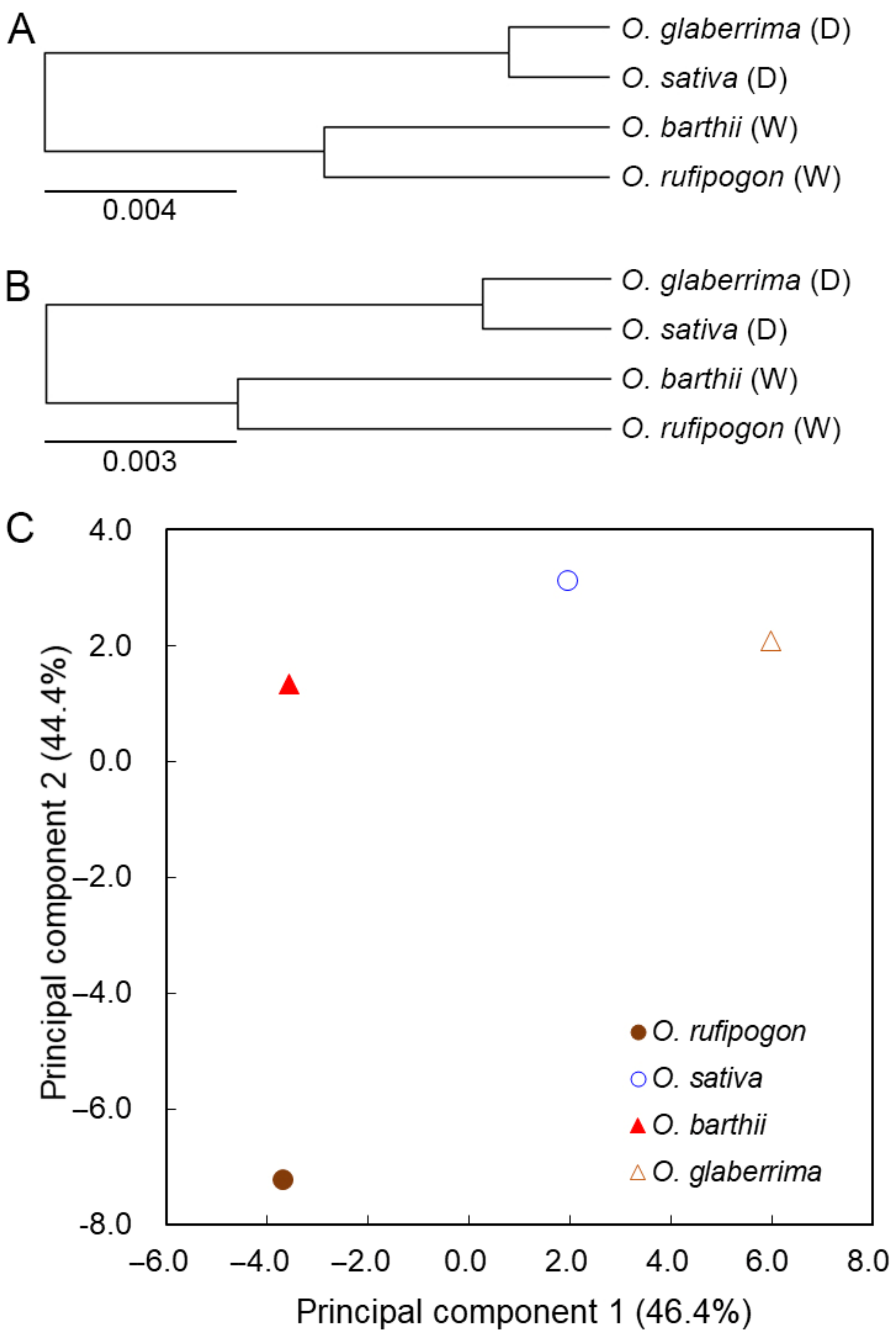

Figure S16. The cluster and principal component analyses using the SCUB frequencies of genes with none to nine introns. A: A cluster tree based on SCUB frequencies. B: A cluster tree based on DNA methylation associated codon combinations. C: The two dimensional scatter plot based on PC1 and PC2 score coefficients.

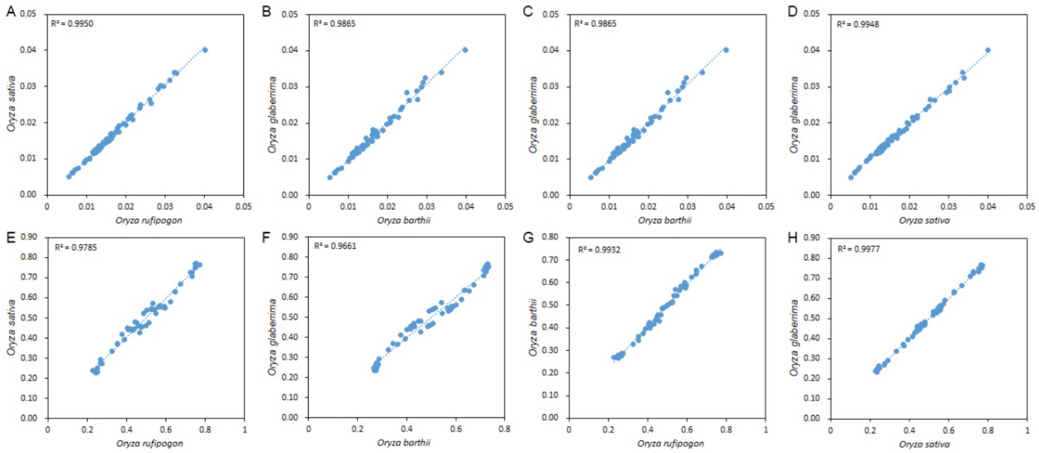

Figure S17. The correlation analysis of SCUB frequencies. A-D: Correlation analysis based on SCUB frequencies of 59 SCs. E-H: Correlation analysis based on SCUB frequencies along exons in genes with two to ten exons. A, E: Asian wild and cultivated rice. B, F: African wild and cultivated rice. C, G: Asian and African wild rice. D, H: Asian and African cultivated rice.
